# Cellular and molecular organization of the *Drosophila* foregut

**DOI:** 10.1073/pnas.2318760121

**Published:** 2024-03-05

**Authors:** Haolong Zhu, William B. Ludington, Allan C. Spradling

**Affiliations:** ^a^Biosphere Sciences and Engineering, Carnegie Institution for Science, Baltimore, MD 21218; ^b^Department of Biology, Johns Hopkins University, Baltimore, MD 21218; ^c^HHMI, Baltimore, MD 21218

**Keywords:** foregut, organ coordination, Drosophila, hormone, digestion

## Abstract

The animal foregut is the first tissue to encounter ingested food, bacteria, and viruses. We characterized the adult Drosophila foregut using transcriptomics to better understand how it triages consumed items for digestion or immune response and manages resources. Cell types were assigned and validated using GFP-tagged and Gal4 reporter lines. Foregut-associated neuroendocrine cells play a major integrative role by coordinating gut activity with nutrition, the microbiome, and circadian cycles; some express clock genes. Multiple epithelial cell types comprise the proventriculus, the central foregut organ that secretes the peritrophic matrix (PM) lining the gut. Analyzing cell types synthesizing individual PM layers revealed abundant mucin production close to enterocytes, similar to the mammalian intestinal mucosa. The esophagus and salivary gland express secreted proteins likely to line the esophageal surface, some of which may generate a foregut commensal niche housing specific gut microbiome species. Overall, our results imply that the foregut coordinates dietary sensing, hormonal regulation, and immunity in a manner that has been conserved during animal evolution.

## Significance

We characterized the adult Drosophila foregut using transcriptomics to provide a genetically tractable model for analyzing how animal foreguts coordinate digestion and immune response with sensory information. Single-cell RNA sequence analysis was strengthened using tagged endogenous genes as reporters to validate candidate cell types and enable cell-type-specific genetic disruption. Neuroendocrine cells coordinate gut activity with nutrition, the microbiome, and circadian cycles. The proventriculus (PV) secretes the peritrophic matrix (PM) that lines the gut, controlling digestion and microbiome interaction. Esophageal and salivary gland–secreted proteins allow us to identify candidate host proteins constituting a foregut commensal niche for specific bacterial species. Overall, foregut structure and function have likely been conserved during animal evolution.

**Fig. 1.**
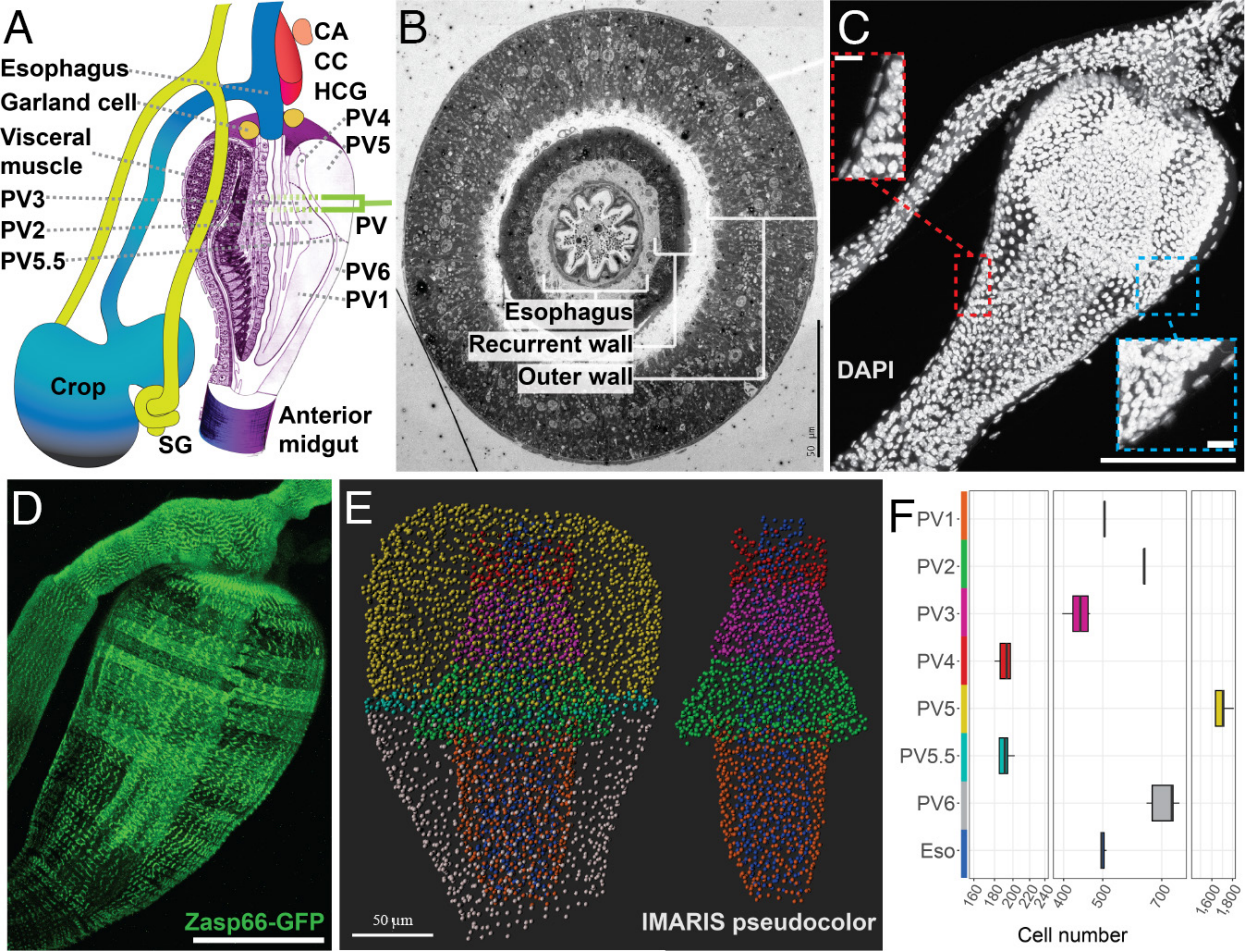
Anatomy of the Drosophila proventriculus and associated foregut tissues. (*A*) Diagram of the Drosophila foregut. The esophagus (Eso), crop, salivary gland (SG), *corpus allatum* (CA), *corpus cardiacum* (CC), hypocerebral ganglion (HCG), Garland cell nephrocyte, and proventriculus (PV) (shown by a cutaway section including the six cell types, PV1-6, modified from 29). (*B*) EM cross-section indicates three cell layers. (*C*) 3D micrograph of foregut nuclei stained by DAPI (white). *Insets*: visceral muscle layer. (*D*) Zasp66-GFP immunofluorescence (green) reveals visceral muscles lining the PV and esophagus. (*E*) 3D micrograph processed using IMARIS (spot function) to segment cell types for automated counting. (*F*) Box plots of indicated cell types and corresponding counts. [Scale bars, 50 µm (*B*); 100 µm (*C*) and 10 µm (*Insets*); 100 µm (*D*); and 50 µm (*E*).]

The foregut is the first tissue to encounter ingested food and potential pathogens entering the animal body. Efficiently extracting and processing consumed nutrients while retaining beneficial microorganisms and neutralizing pathogens represents a major challenge for this gatekeeper tissue. These tasks are accomplished in insects such as *Drosophila melanogaster* by a complex of cellular structures collectively termed the foregut (Fig. 1*A*) located in the head and thorax (1). Entering food and microorganisms move into the esophagus and crop duct where some beneficial symbiotic bacteria bind and proliferate (2–5). Host factors that regulate and maintain specific bacterial colonizers are currently unknown.

The esophagus directs food into the proventriculus (PV), the central foregut organ, whose valve-like action regulates midgut entry [(1, 6); Fig. 1*A*]. Prior to passage into the intestine, food is mixed with salivary gland (SG) secretions and may be diverted into the crop, an expandable muscular sack for temporary storage and preprocessing (1, 7). Mammalian intestinal epithelia are covered by a mucosal layer that serves as a barrier (8). The Drosophila PV synthesizes a major digestive product, the multilayered peritrophic matrix (PM) that likewise remains between consumed items and enterocytes as food passes through the gut (9). The foregut epithelium and the PM restrict pathogens, promote digestion, and provide additional physiological benefits (10–12).

The information on which the foregut bases its decisions as a gatekeeper is received from diverse neural connections and from other tissues that report nutritional and immune status. In most animals, the foregut's outputs are transduced by neurosecretory cells that secrete peptide hormones received by epithelial and gut targets (13, 14). Hormone-secreting neurosecretory cells reside in glands, including the *corpus allatum* (CA) and *corpus cardiacum* (CC), which associate with the esophagus and hypocerebral ganglion (HCG) in the anterior foregut while retaining neural communication with the brain [(15); Fig. 1*A*].

The CA is the major source of juvenile hormone (JH), named for its roles regulating metamorphosis [review: (16)]. In adults, JH produced in the foregut controls nutrient-sensitive processes including yolk production, reproductive diapause, and aging (17–19). Moreover, JH regulates PM production by the *Calliphora erythrocephala* PV (20). Multiple proteins with JH–binding domains are expressed in the gut, suggesting that JH is broadly involved in regulating gut function (21).

CC cells (Fig. 1*A*) produce the glucagon-like adipokinetic hormone (Akh), which mobilizes fat and carbohydrates stored in fat cells and other sites (22). The CC transduces information from the nervous system and from conserved enteroendocrine peptides produced in the gut to coordinate metabolic activity with varying external conditions (23, 24). A small number of nephrocytes (garland cells) also associate with the anterior region of the PV surrounding the esophagus (25, 26).

The foregut develops in the embryo from both ectoderm as well as endoderm cells generated during gastrulation. Part of the primitive gut tube including the ectoderm–endoderm junction undergoes involution to produce the three PV layers: the outer wall, recurrent wall, and central esophagus [Fig. 1*B*, (27, 28)]. The cellular makeup of the adult Drosophila proventriculus was described by King (29), who identified 6 zones containing distinct cell types we refer to as PV1-6. Electron microscopy revealed four PM layers (L1 to L4), two of which could be seen to arise from specific PV cells.

This characterization of the Drosophila foregut at single-cell resolution, combined with previous studies of the midgut (30, 31), Malpighian tubules (32, 33), and hindgut (25) now brings nearly an entire animal digestive system to a cellular level of understanding. Our study suggests that the foregut, by virtue of its location near the brain and sensory organs and the most frequent site of pathogen invasion—the esophagus, acts as a central coordinator of intestinal and immune activities. By characterizing foregut cell types, inferring their biological roles, and identifying tools to query their gene functions, this work advances understanding of the foregut’s important and conserved roles.

## Results

### Taking a Census of Proventricular Cells Using High-Resolution Microscopy.

We counted the number of PV cells to assist in recovering equally each cell type. Because of its involution during development, the PV comprises three cell layers rather than a simple tube (Fig. 1*B*). A peripheral layer of surrounding visceral muscle sheath (VM) was also evident from their elongated nuclei (Fig. 1 *C*, *Insets*). VM cells were revealed in detail using Zasp66-GFP expression (Fig. 1*D*), and PVs cultured briefly in vitro pulsated in rhythmical waves (Movie S1). Three-dimensional (3D) light microscopy and IMARIS software (*SI Appendix*, *Supplemental Methods*) allowed us to determine how many cells make up each of the PV epithelial cell zones (Fig. 1 *C*, *E*, and *F*). In addition to the PV1-6 regions, we observed a small zone of denser cell nuclei indented into the outer wall between PV5 and PV6. These cells are shown below to comprise a previously undescribed cell type we term “PV5.5.” Overall, an average of 190 to 1,660 cells reside within each cell zone (Fig. 1*F* and *SI Appendix*, SI Dataset, Table S1).

### Analysis of Foregut Cells Using scRNAseq and In Vivo Gene Expression Reporters.

**Fig. 2.**
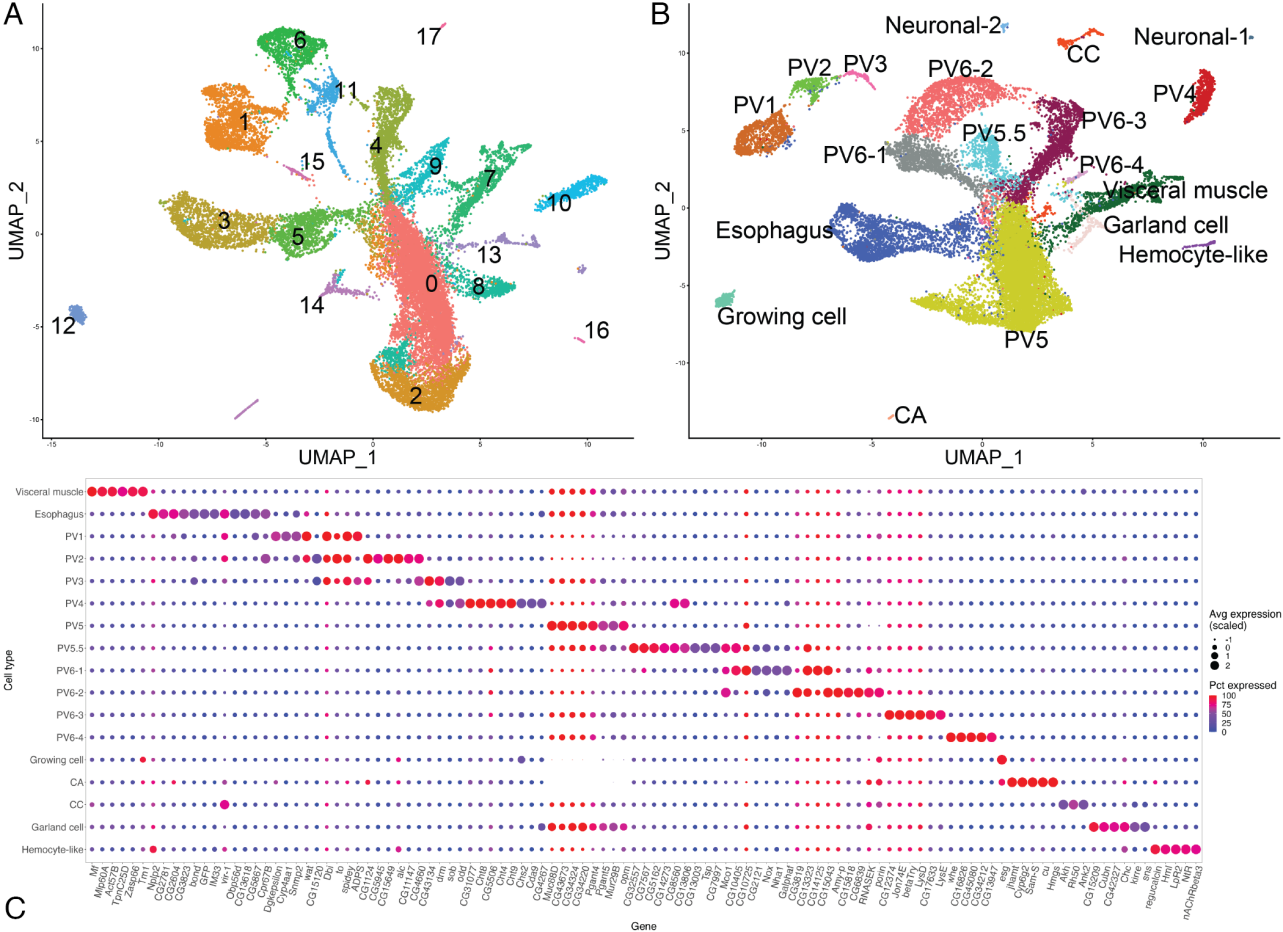
Identification and clustering of major foregut tissue cell types by scRNAseq. (*A*) UMAP plot of the initial scRNAseq data with 18 putative cell clusters. (*B*) UMAP plot of scRNAseq data refined to resolve 19 cell types based on gene expression and reporter lines. Cell groups are colored and labeled. (*C*) Expression levels and uniformity in the indicated cell clusters (*y*-axis) of selected genes (*x*-axis) that can serve as markers of foregut cell types.

We analyzed the cell-type-specific gene expression of Drosophila foregut tissues using single-cell RNA sequencing (scRNAseq) (Fig. 2 and *SI Appendix*, Fig. S1). To promote recovery of an unbiased cell sample, we manually unfolded the triple-layered PV prior to scRNAseq library construction (*SI Appendix*, Fig. S1 *A* and *B* and *Supplemental Methods*). We analyzed three replicate datasets using Cell Ranger and Seurat (34). The initial analysis placed 21,948 high-quality cells in 18 cell clusters on a UMAP plot (Fig. 2*A*). To correlate cell clusters with cell types, we identified sets of genes that are specifically expressed in each cluster (*SI Appendix*, Fig. S1*C*). For example, cluster 7 expressed muscle-related genes, cluster 13 showed neuronal features, and cluster 16 expressed genes involved in JH synthesis. A large group of clusters near the center of the UMAP expressed genes associated with epithelia, and some of these cells expressed known PV genes including *Muc68D* and *Pgant4*.

**Fig. 3.**
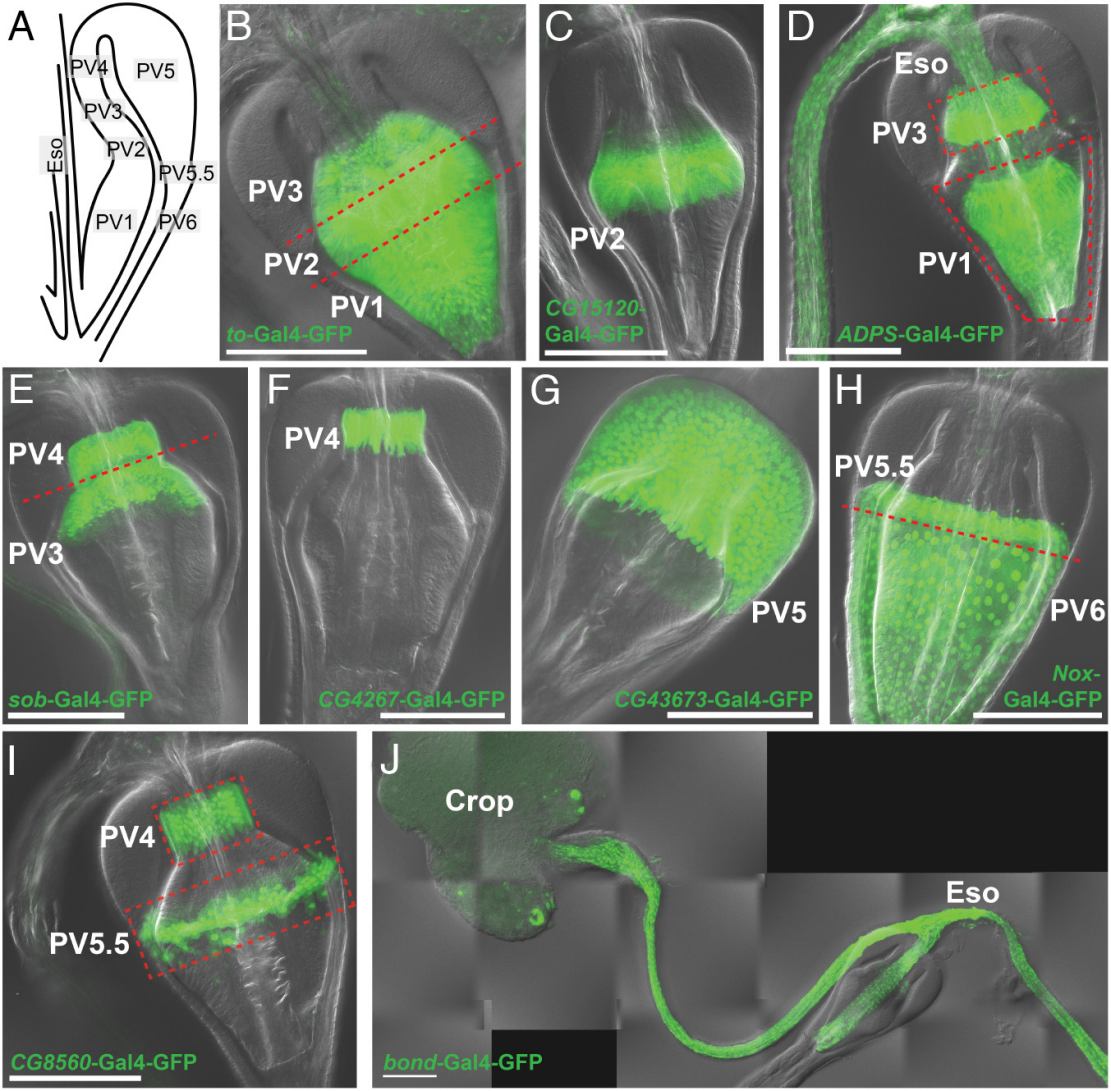
Annotation and validation of PV cell clusters using marker gene reporters. (*A*) Schematic of PV1-6 and esophagus within PV. (*B*) *to*-Gal4-GFP labels PV1-3; (*C*) *CG15120*-Gal4-GFP labels PV2; (*D*) *ADPS*-Gal4-GFP labels PV1, PV3, and Eso; (*E*) *sob*-Gal4-GFP labels PV3, and PV4; (*F*) *CG4267*-Gal4-GFP labels PV4; (*G*) *CG43673*-Gal4-GFP labels PV5; (*H*) *Nox*-Gal4-GFP labels PV5.5, and PV6; (*I*) *CG8560*-Gal4-GFP labels PV4, and PV5.5; (*J*) *bond*-Gal4-GFP labels Eso. Green = GFP signal. (Scale bar, 100 µm.)

Individual cell clusters were further analyzed using more than 150 gene reporter lines (*SI Appendix*, SI Dataset, Table S2) mostly generated by the FlyTrap Project (35, 36) or the Gene Disruption Project (37). Reporters for candidate genes specific to individual cell clusters (Fig. 2*A* and *SI Appendix*, Fig. S1*C*) were analyzed directly or after crossing to UAS-GFP to identify which cell types they label. For example, expression of *takeout (to)* highlighted PV1-3 (Fig. 3 *A* and *B*), *CG15120* labeled PV2 (Fig. 3*C*), *ADPS* labeled PV1, PV3, the esophagus and the crop duct (Fig. 3*D*), *sob* labeled PV3 and PV4 (Fig. 3*E*), *CG4267* labeled PV4 (Fig. 3*F*), *CG43673* labeled PV5 (Fig. 3*G*), *Nox* labeled PV5.5 and PV6 (Fig. 3*H*), CG8560 labeled PV4 and PV5.5 (Fig. 3*I*), while *bond* labeled the esophagus and crop duct (Fig. 3*J*). The crop duct co-labeled with the esophagus by all gene reporters we examined, suggesting that it is an esophageal extension.

We further refined the clustering to better match the gene expression data by varying several analytic parameters in Seurat. In several cases, such as PV2 and PV3 cells, which initially coclustered, separation into individual cell types was achieved with subclustering analysis. In summary, we identified 19 cell clusters including the 7 PV epithelial cell types described by King (Fig. 3), the PV5.5 cell type, and other PV-associated cell types (Fig. 2*B*). Final normalized gene expression in these cell clusters (*SI Appendix*, SI Dataset, Table S3) allowed 24 to 794 genes preferentially expressed within each of the 19 clusters to be identified (*SI Appendix*, SI Dataset, Table S4 and Fig. 2*C*).

### Development of a Toolkit for Foregut Cell-Type-Specific Gene Expression Control.

Cell-type-specific drivers provide a powerful tool for studying gene and cell functions since they can be used to overexpress genes or knock down genes via RNAi. Our studies identified many Gal4 driver or GFP tag lines preferentially expressed in foregut cell clusters that will be useful for analytical purposes (*SI Appendix*, SI Dataset, Table S5).

### PV Epithelial Cells Build a Stratified PM.

**Fig. 4.**
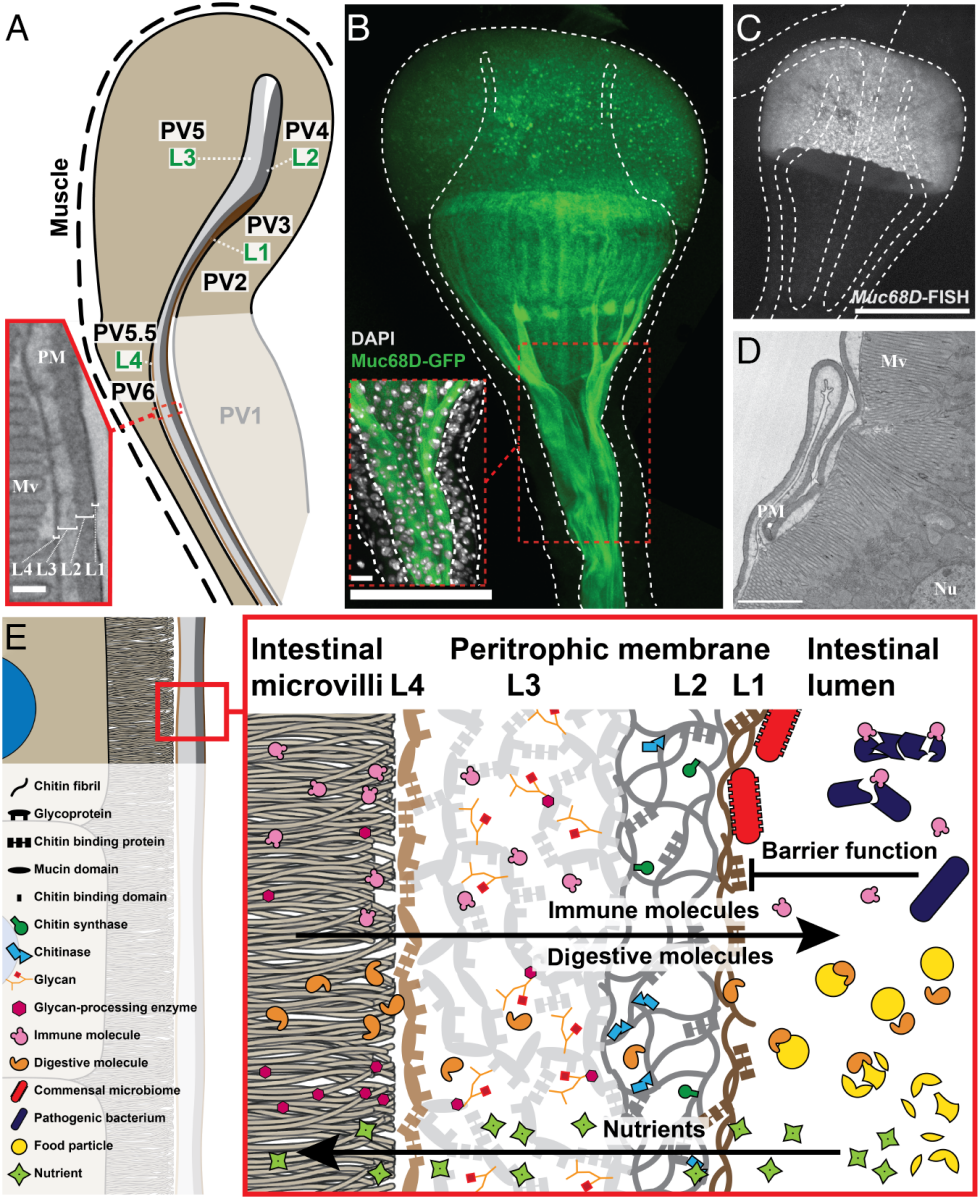
PV cells produce a multilayered PM. (*A*) The electron microscopic diagram (*Inset* at the *Left*) reveals the four PM layers (L1 to L4) corresponding to PM production of L1 from PV3, L2 from PV4, L3 from PV5, and L4 from PV5.5 and PV6. (*B*) Muc68D-GFP fusion staining (green) shows PM production in PV5 cell secretory vesicles, Muc68D protein in secreted PM moving toward the midgut and converging to form the completed PM. *Inset*: PV diameter reduction may induce PM buckling. (*C*) RNA-FISH (white) documents expression of *Muc68D* transcripts only in PV5 cells. (*D*) EM section of posterior PV shows PM structure and buckling. (*E*) Model of layered PM structure (L1 to L4) based on these studies (see key defining symbols on *Left*). Microvilli (Mv), and nucleus (Nu). [Scale bars, 0.2 µm (*A*); 100 µm (*B*), 20 µm (*Inset*); 100 µm (*C*); and 2 µm (*D*).]

One major function of the PV is to produce the PM that encloses food and moves through the GI tract. Previous biochemical studies revealed that the PM is highly enriched in chitin and glycoproteins, structures able to resist active digestive enzymes (6, 9). Electron microscopic studies of the PM identified four layers called L1 to L4 [Fig. 4*A*; (20, 29)]. The two most anterior cell types, PV4 and PV5, first secrete the middle layers, L2 and L3, respectively. The L1 layer appears after the bilayer reaches PV3. As the PM moves posteriorly, the L4 layer appears when the layers reach PV5.5 and PV6. We sought information about PM structure by examining preferentially expressed genes in PM layer–producing cells.

The L1 and L2 layers are electron-dense (Fig. 4*A*) and likely serve as a barrier between the gut contents and the host (Fig. 4*E*). The large L2 layer is almost certainly enriched in chitin polymers, since its cell of origin, PV4, specifically expresses *chitin synthase* 2 (*Chs2*), encoding an ortholog of the enzyme used to synthesize PM chitin in previously investigated insects (38, 39). PV4 cells also produce seven mRNAs encoding proteins containing chitin-binding domains (CG31077, Cht8, Cht4, Cht9, CG13806, Idgf3, and Cht5) which may bind or modify chitin polymers to incorporate immune and digestive proteins (40). We conjecture that L1 is secreted by PV3 cells and associates with the chitin-rich L2 layer after the initial PM moves posteriorly to overlie these cells. Some of the most abundant PV3-enriched mRNAs encode secretory proteins consistent with such a function. These include putative structural proteins with chitin-binding domains including CG3348, Idgf6, Idgf4, dpy, Idgf3, and pio. These molecules might help attach L1 to the L2 layer. We also detected preferential expression of other secretory molecules from PV3 such as the JH-binding protein gene *Jhbp1* and the related protein *takeout* (*to*).

The middle PM layer, L3, occupies most of the PM volume and is produced by the large and abundant PV5 cells in the anterior PV. PV5 expresses mucin genes, including the abundantly expressed *Muc68D* as well as *Mur29B*, which show sequence and structural features similar to mucin genes in other species (41). Mucins contain tandem repeats rich in proline, threonine, and/or serine (PTS repeats) and are highly glycosylated (41, 42). We validated the contribution of Muc68D to PM formation by generating a Muc68D-GFP reporter fly line and visualizing its expression (Fig. 4*B*). *Muc68D* RNA was only significantly detected in PV5 based on RNA-fluorescence in situ hybridization (Fig. 4*C*). In contrast, labeled Muc68D protein is apparent as cytoplasmic vesicles (Fig. 4*B* and *SI Appendix*, Fig. S2*A*) that are secreted into the extracellular space where they coalesce into a sheet that passes into the posterior PV and then the anterior midgut (Fig. 4*B*). As the PM moves posteriorly, it bulges and sometimes folds into the luminal space (Fig. 4 *B* and *D* and *SI Appendix*, Fig. S2*D*). The diameter of the PV housing the PM decreases from about 100 µm to 50 µm in its posterior region as it approaches the midgut (Fig. 4*B*), which may drive PM buckling (Fig. 4 *B* and *D* and *SI Appendix*, Fig. S2*D*).

Glycosylation of proteins makes them more resistant to digestive enzymes and is a common feature in the GI tract of many organisms [review: (41)]. PV5 expresses multiple glycosylation genes, including *Pgant 4*, *Pgant 5,* and *Glucuronyltransferase P* (*GlcAT-P*). Genes are also expressed encoding O-glycan and N-glycan-processing enzymes, including hexosaminidase 1 (*Hexo1*), glucosamine-phosphate N-acetyltransferase (*Gnpnat)*, glutamine:fructose-6-phosphate aminotransferase 1 (*Gfat1*), and glycoprotein-N-acetylgalactosamine 3-beta-galactosyltransferase (*tgy*), as well as N-acetylgalactosaminide beta-1,3-galactosyltransferase (*CG34452*), and UDP-glucose 6-dehydrogenase (*sgl)*. Protein secretion and glycosylation in L3 are further borne out by PV5-expressed Golgi-focused secretory pathway genes including *sauron* (*sau*), *opossum* (*opm*), and *CG33298* and by expression of the transcription factor *CrebA* (43) which up-regulates the canonical secretory pathway (Fig. 4*E*).

Our results also clarify the origin and nature of the PM L4 layer. The previously unrecognized PV5.5 cells occur where L4 first appears, and PV5.5 gene expression includes several chitin-binding proteins (CG13806, CG14300, CG13003, and Cpr51A). PV6 gene expression specifies additional proteins with chitin-binding domains (CG14125, CG10725, and Cpr51A), some of which are also expressed by PV5.5. These gene expression profiles suggest that L4 differs from L1 by encoding ECM-like proteins mediating interactions with enterocytes (Fig. 4*E*).

### Posterior PV Cells Connecting with the Anterior Midgut Produce Digestive Enzymes.

King’s PV6 zone comprised the posterior half of the PV outer wall including the zone where the PM is loaded with esophageal contents before they pass into the anterior midgut (*SI Appendix*, Fig. S3*A*). Our scRNAseq resolved this large region into four cell clusters, PV6-1, PV6-2, PV6-3, and PV6-4 (Fig. 2*B*). The most abundant genes expressed by PV6-2 comprise secreted digestive enzymes (*SI Appendix*, SI Dataset, Tables S3 and S4). These include *Amy-p*, which encodes an amylase that is orthologous to human pancreatic amylase Amy2B and mouse Amy1A. *CG3819*, *CG6839,* and *CG33346* are related, abundantly expressed genes predicted to encode proteins with RNase and single-stranded DNase activities. PV6-2 cells also express *Phae2* which encodes a serine endopeptidase of the S1A chymotrypsin family that is closely related to kallikrein1 (KLK1), a secretory product in the human esophagus and the pancreas (44).

The PV6-3 cell group also contributes to digestive enzyme production. Abundant predicted products include carboxypeptidase CG12374, metalo-carboxypeptidase CG17633, serine hydrolases Jon74E, Jon99Cii, Jon65Aiii, and Jon65aiv, and trypsins alphaTry, betaTry, kappaTry and epsilonTry, along with more than 10 additional serine hydrolases. Along with the digestive enzyme genes, PV6-3 cells additionally express three mucin genes, *Muc68E*, *Muc14A,* and *Muc55B*. These observations suggest that an initial supply of digestive enzymes is produced in the posterior PV, where they become associated with the PM wall or are deposited into the intestinal lumen along with the esophageal contents.

### Gene Expression and Function of the Esophagus.

**Fig. 5.**
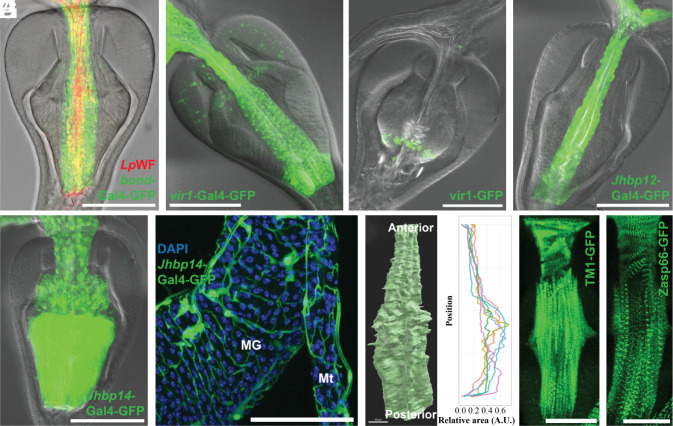
The esophagus and its anterior valve. (*A*) PV esophagus core labeled by *bond*-Gal4-GFP (green). Colonization in foregut commensal niche of fluorescently labeled *L. plantarum* (*LpWF*) (red) within the Eso. (*B*) *vir1*-Gal4-GFP expression (green) in the Eso. (*C*) vir1-GFP-tagged expression (green) in the extracellular space between esophagus and PV1 region. (*D*) *Jhbp12*-Gal4-GFP (green) in the Eso. (*E* and *F*) *Jhbp12*-Gal4-GFP (green) within the Eso, PV1-3 (*E*) and tracheoles (*F*) surrounding the midgut (MG) and Malpighian tubules (Mt). (*G*) X-ray microcomputed tomography (XR µCT) [*Left*, (4)]; analysis (*Right*) quantitates longitudinal size expansion within PV esophagus core. (*H* and *I*) Tm1-GFP (*H*) and Zasp66-GFP (*I*) (green) reveal longitudinal muscle fibers of the Eso and asymmetric fibers at its anterior that are proposed to function as a valve. [Scale bars, 100 µm (*A*–*F*); 30 µm (*G*); and 50 µm (*H* and *I*).]

The esophagus connects to the posterior GI tract by passing through the PV as its inner wall. We identified the esophagus cell cluster by its preferential *bond* expression (Fig. 3*J*). Esophageal cells produce many mRNAs that are shared with ectoderm-derived PV1-3 cells but differ more extensively from outer wall cells, which are of endodermal origin. Many abundant esophageal and PV1-3 genes encode proteins known or predicted to play roles in fatty acid (FA) metabolism. For example, acetyl coenzyme A synthase (AcCoAS), Acetyl-CoA carboxylase (Acc), Fatp, Fasn1, and CG8306 are involved in FA synthesis. Hacd1 is involved in FA elongation and sphingolipid synthesis, while bond, CG2781, Baldspot, Sc2, and CG5362 contribute to long-chain FA production. FA reductase CG5065 likely generates waxes, while CG34120 and CG3823 are involved in transmembrane lipid transport. Fat metabolism has been genetically linked to survival after virus infection (45), suggesting that the extensive lipid metabolism of esophageal and PV1-3 cells serves immune functions. Recent work shows that the Drosophila esophagus contains a foregut commensal niche that houses specific microbiome species including *Lactiplantibacillus plantarum* (*LpWF*) [(4); Fig. 5*A*]. Therefore, the esophagus must allow symbiotic bacteria to colonize the epithelium, while still protecting the host from pathogen invasion.

Consistent with this idea, several known immunity genes are strongly expressed by esophageal cells, including *vir-1*, *Nplp2*, *vvl*, *CG17919,* and *EbpIII*. *vir-1* transcription is induced upon viral infection, possibly in response to Jak/STAT signaling (46). We observed high *vir-1* expression in esophageal cells compared to several other PV cell types (*SI Appendix*, Table S4), in agreement with the *vir-1* gene trap line (Fig. 5*B*). Furthermore, we generated a vir-1-GFP line starting with a MiMIC insertion in the first *vir-1* coding intronic region [(37); *SI Appendix*, *Supplemental Methods*]. Interestingly, vir-1-GFP accumulated in cells and extracellular space between the esophagus and the posterior recurrent wall (Fig. 5*C*), suggesting an immune function in this region.

Another group of proteins expressed in esophageal cells include Jhpb12 (CG13618), Jhbp7 (CG11852), and Jhbp14 (CG5867), which are part of the 16-member family of JH binding proteins. *Jhbp12* expression revealed by the CRIMIC line CR02124 (47) agrees with scRNAseq analysis that finds *Jhbp12* preferentially expressed in the esophagus (Fig. 5*D*). *Jhbp14* production, as visualized by CR02482, also shows esophageal activity, as well as expression in PV1-3 (Fig. 5*E*). Moreover, *Jhbp14* is expressed in some tracheoles wrapping around the midgut and Malpighian tubules (Fig. 5*F*). Other members of the Jhbp family are also found in foregut cells including *Jhbp1* (PV2, PV3, and CA), *Jhbp13* (PV2), and *Jhbp3* (PV1, PV3). The function of JH-binding proteins, which share high-affinity JH-binding domains, is not well characterized. These cells may carry out functions mediated by JH binding beyond those controlled directly by the canonical JH receptors.

The luminal space of the esophagus within the PV comprises a narrower anterior segment and a wider posterior portion as revealed by X-ray microcomputed tomography (Fig. 5*G*). Like other ectodermally derived cells, the esophageal and PV1-3 cells secrete a cuticle apically (29). Three known cuticle genes, *Cpr67B*, *Cpr49Ae*, and *Cpr51A*, are preferentially expressed in these cells. This cuticle layer folds into longitudinal crypt-like furrows [(4); Fig. 1*B*] which may contribute to its ability to specifically associate with some bacteria.

### The Nature of the Proventricular Valve.

The PV has been classically described as a valve that restricts flow into the intestine by constricting the esophagus within its middle region. By live imaging of freshly dissected tissue, we were able to visualize contractions of the PV that are consistent with the valve hypothesis (Movie S1). To examine the esophageal musculature in greater detail, we used the Tm1-GFP (Fig. 5*H*) and Zasp66-GFP (Fig. 5*I*) fly lines. In the anterior esophagus near its point of entry to the PV, we observed angled circumferential visceral muscle fibers surrounding the esophageal tube that might be able to pinch off flow through the lumen. We propose that this twisted musculature structure may be the Drosophila pharynx that controls the ingestion into the intestine. In addition, we observed longitudinal visceral fibers that extend along much of the length of the esophageal tube, and these may contribute to its peristaltic contractions (Movie S1). These observations suggest that the PV valve occurs at the anterior end rather than in the medial part of the PV as previous studies suggested (29).

### The Foregut Neurosecretory Glands.

The adult CA, CC and associated neurosecretory cells anterior to the PV [(15); Fig. 1*A*] comprise the major endocrine tissues of the foregut responsible for its function as a metabolic and reproductive regulatory axis. They arise during pupal development from the larval ring gland but lack a known analog of the larval ecdysone-producing prothoracic gland (48). Our studies provide gland-specific transcriptome information on these important tissues. scRNAseq analysis of the CA cluster identified 393 preferentially expressed genes (*SI Appendix*, SI Dataset, Table S4, CA), whose nature left no doubt about cluster identification. The two most abundant CA genes were *jhamt* encoding JH acid methyl transferase (catalyzing the final step in JH biosynthesis) and *Cyp6g2* which is also required for JH synthesis and may encode the penultimate step (48). At least five of the seven enzymes in the mevalonate pathway leading to the JH precursor farnesoic acid were also among the highest expressed CA-specific genes. Metascape analysis (49) showed the CA cluster preferentially expresses genes in additional pathways. Most striking among these were at least 19 circadian clock genes (*P* < 1.0E^−9.2^), encoding clock components, *timeless* (*tim*), *clockwork orange* (*Cwo*), as well as *cryptochrome* (*cry*) which entrains the clock to circadian light changes. Other highly expressed circadian genes were *lark*, clock-interacting protein (*Cipc*), *Ahcy*, *curled*, *Hmgcr*, and at least 14 genes contributing to the proteosome, the cellular machine that degrades ubiquitin-modified proteins and plays a key role in circadian timekeeping. Many other canonical clock genes were also expressed more highly in the CA than in other foregut tissues.

**Fig. 6.**
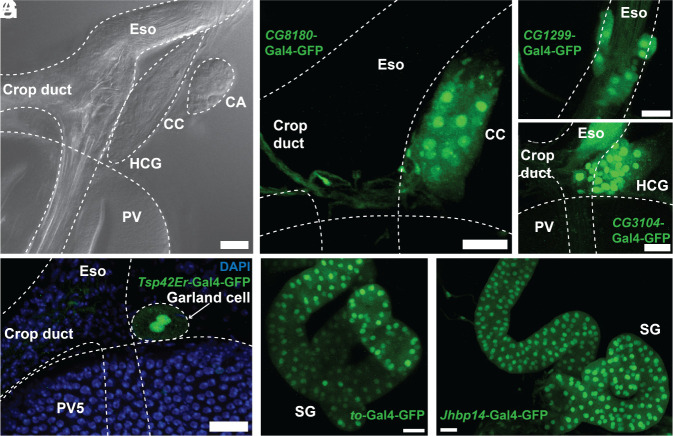
Foregut neural, glandular, and secretory cells. (*A*) Light micrograph showing the CA, CC, and HCG attached to the Eso near its junction with the crop duct. (*B*) *CG8180*-Gal4-GFP (green) labels CC and associated nerves near the Eso-crop duct junction. (*C* and *D*) Eso region near crop duct junction showing expression of *CG1299*-Gal4-GFP (green) (*C*), or *CG3104*-Gal4-GFP (green) (*D*). (*E*) A binucleated garland cell nephrocyte (arrow) highlighted by *Trp42Er*-Gal4-GFP (green). (*F* and *G*) SG labeled by *to*-Gal4-GFP (green) (*F*), or *Jhbp14*-Gal4-GFP (green) (*G*). (Scale bar, 20 µm.)

The second major neuronal endocrine organ attached to the esophagus is the CC (Fig. 1*A*). Among 396 preferentially expressed CC genes (*SI Appendix*, SI Dataset, Table S4, CC), *adipokinetic hormone* (*Akh*) was the most highly expressed protein-coding gene (22). Metascape analysis of preferential CC genes highlighted many genes and Metascape categories related to neural development and synaptic transmission. A GFP reporter for the CC gene *CG8180* labeled CC cells (Fig. 6*B*). Anatomically, the CC is known to connect with the central nervous system, with the adjacent, HCG, and with axons projecting posteriorly onto the PV, the crop stalk, and the SG (15).

Further insight into the clustered adult neuroendocrine organs associated with the esophagus came from analysis of the Neuronal-1 (N1) cell cluster (*SI Appendix*, SI Dataset, Table S4, N1). The results suggested that this cluster includes several different types of neurosecretory cells, possibly located within the HCG and associated neurons, consistent with the observed expression of the N1 reporter *CG3104* (Fig. 6*D*). The most abundant preferentially expressed N1 cluster gene encodes the neuropeptide CCHa2, a signal produced by fat cells and gut enteroendocrine cells that is thought to regulate appetite and insulin-like peptide production (50). The second most abundant gene encodes tachykinin (Tk), a neuropeptide known to influence gut motility. Tk produced by neurons within the CC/HCG that exit posteriorly may contribute to rhythmic PV contractions. Many other N1 genes encode proteins that process and modify neuropeptides (*SI Appendix*, SI Dataset, Table S4, N1).

The growing cell scRNAseq cluster is also likely to promote neuroendocrine function (Fig. 2*B*). Metascape analysis (*SI Appendix*, SI Dataset, Table S4, Growing cell) identified highly significant GO processes including “generation of neurons, asymmetric cell divisions, neuroblast proliferation, and asymmetric neuroblast proliferation.” These results suggest that neurogenesis is ongoing in the foregut in support of the adult neuroendocrine axis.

### Garland Cell Nephrocytes Express Genes Involved in Filtration.

We observed Garland cell nephrocytes, recognizable by their large size, location at the entrance of the esophagus into the PV, and binucleated morphology as highlighted in *Trp42Er*-Gal4-GFP flies (Fig. 6*E*). Nephrocytes contain conserved structures related to the slit diaphragms of mammalian glomerular podocytes (51, 52). The Garland cell cluster expressed the mammalian *Neph1* ortholog *kirre* and the *Nephrin* ortholog *sns*. Metascape analysis identified highly significant GO terms associated with this cluster including “nephrocyte filtration” and “garland nephrocyte differentiation.” Additional likely garland cell genes include *Mec2* (orthologous to human *stomatin*) a likely slit diaphragm component, *amnionless* (orthologous to human *AMN*), *C1C-c*, *CG30344* (orthologous to human *SLC46A1* and *SLC46A1*) which promote renal tubular secretion, as well as *Cubn2* (orthologous to human *cubilin*), and *Cubn*, which are part of a receptor complex involved in renal protein absorption by larval garland cells. Many other genes specifically expressed in this cell group are likely to be important for adult renal glomerular development and function, including *Hand*, *Obp18a*, *CG15209,* and *CG9953* (orthologous to mammalian *Prss16*).

### The Crop and SG.

Two foregut-associated tissues, the adult SG and the crop, were not present in the material used for scRNAseq. To provide a resource for studying these tissues, we separately dissected crops and SGs from adult female flies and carried out RNAseq (*SI Appendix*, SI Dataset, Table S6). The crop temporarily stores ingested food before returning it to the esophagus for digestion [(1); *SI Appendix*, Fig. S4]. One of the crop’s most highly expressed genes, *Alcohol dehydrogenase* (*Adh*), plays a protective role when adults feed on fermenting fruit (53). In the human esophagus, *alcohol dehydrogenase 7* is highly expressed and differentially up-regulated compared to other tissues (44), suggesting a conserved foregut function. Crop gene expression resembled other visceral epithelial cells, but we did not observe notable digestive enzyme expression.

The SG was found to produce a few enzymes at relatively low levels, including Idgf3, which might degrade chitin, DNaseII, Adh, and Mal-B1 maltase. In addition, it produces several products potentially involved in resistance to bacterial pathogens (lysozyme LysP, carbonic anhydrase CAH1, innate immune activator mtd), the water channel Drip, and enzymes such as esterase 6 (Est-6) with no known digestive role. Multiple drivers including *to*-Gal4-GFP (Fig. 6*F*) and the *Jhbp14*-Gal4-GFP (Fig. 6*G*) labeled SG secretory cells.

### Immunity Genes Are Induced in Axenically Raised Adults upon Exposure to a Normal Commensal Bacterial Species.

To investigate immunity in the PV under controlled microbial conditions, we performed bulk RNA sequencing on dissected PVs from axenic flies and flies associated with the normal commensal bacterial species *LpWF*. We found that the introduction of *LpWF* increased the expression of immune genes in the PV compared to the axenic control (*SI Appendix*, Fig. S3*C* and Table S7). These genes include antimicrobial peptides *DptB*, *AttB*, *AttA*, and *Def* as well as other immune molecules such as *IM18*, *PGRP-SD*, *PGRP-SB1*, and *pirk*. This raises the question of whether development in the presence of commensals causes an adaptation that diminishes a defensive reaction to these species (54).

## Discussion

### The Cellular and Genetic Makeup of an Animal Digestive System Is Now Largely Complete.

The addition of the Drosophila foregut at single-cell resolution to previous studies of the midgut (25, 30–33) and hindgut (25) brings nearly an entire animal digestive system to a cellular level of understanding. Most individual gut cell types and cell-type enriched gene expression have now been characterized. The adult foregut was the last gut region to be analyzed at this depth, probably because of its complexity and diversity compared to other gut regions. Broad surveys of animal cell types, often made using whole animals, are worthwhile but cannot currently substitute for the in-depth analysis of individual cell types as reported here. We ensured that cells were recovered in amounts similar to their representation in vivo (*SI Appendix*, SI Dataset, Table S1), which is impacted by size, shape, adhesivity, and tissue location. We validated (or contradicted) conclusions about cell types and tissue locations suggested by bioinformatic analysis using gene fusions.

Our studies strongly support previous studies of the foregut as a gatekeeper and central coordinator of gut activity with tissues such as the ovary requiring nutrition-dependent resources. By determining individual transcriptomes of the major cell types comprising the Drosophila adult neurosecretory axis, including the CA, CC, HCG, associated neural cells, and garland cells, we gained a more complete picture of their capabilities, clustered on a small region of the esophagus. It will now be easier to study how diet, stress, infection, aging, and other variables affect the intestinal tract and hormonal axis under conditions where the complexities of intertissue interactions can be identified, understood, and genetically dissected.

### The CA Mediates Circadian Gene Expression and Behavior.

Recent studies provide instructive examples of foregut-mediated tissue integration. Drosophila in temperate regions decrease JH expression in response to seasonal reductions in day length and temperature. As a result, the ovaries and gut enter reproductive dormancy, which greatly reduces egg production and digestive activity (17, 19). Brain neurons that project to the CA sense seasonal change and control the onset of reproductive dormancy by producing the neuropeptide Dh31. CA-associated Dh31 receptor (Dh31-R) activation then triggers cAMP elevation and represses JH production (55). In our experiments, CA cells preferentially express *Dh31-R*, which is orthologous to the human calcitonin receptor, but not *Dh31*. At least 36 GFP reporter lines and Gal4 lines from the CRIMIC and MiMIC collections that drive genes preferentially in the CA compared to other foregut tissue were identified that will facilitate further dissection of CA functions (*SI Appendix*, SI Dataset, Table S5).

The finding that the CA cluster expresses circadian genes raises the question of whether circadian cells are distinct from JH-producing cells. If so, individual cells within the cluster should differ in their expression of circadian and JH-production markers. Circadian cells can be recognized by their high expression of the *Ahcy* gene encoding Adenosylhomocysteinase, a regulator of chromatin methylation reactions at circadian target genes in mammals (56), that is also found in Drosophila circadian neurons (57). *Ahcy* ranked among the top seven preferentially expressed CA genes along with *jhamt* and *Cyp6g1* (*SI Appendix*, SI Dataset, Table S4, CA). Another methylation-promoting gene, *Sam-S* encoding S-adenosylmethionine synthetase, was also highly and specifically expressed, suggesting that it also supports circadian function. The JH pathway genes *jhamt* and *Cyp6g1* and the circadian genes *Ahcy* (and *Sam-S*) were highly expressed in all CA cells (Fig. 2*C*, row “CA”). These findings argue that a single CA cell type functions in both hormone production and as a local circadian clock.

Another circadian behavior controlled by the CA is summiting (58). This programmed response elicited by various pathogens requires DN1p circadian neurons, neural inputs from the pars intercerebralis (PI), and the CA itself. In this case, PI to CA neurons highlighted by the driver Gal4 line R19G10 were shown to extensively synapse on the esophagus near its junction with the PV, and with the CA (58). We observed that N1 cells are specifically enriched in AstC-R2, the receptor for the neuropeptide AstC whose production by DN1p circadian neurons is critically important for oogenesis rhythms (59).

Our transcriptome of the adult CA reveals that the *esg* gene is highly expressed. This suggests that the gut regression observed after knocking down *jhamt* expression using esg-Gal4 (60) is likely due to loss of JH expression in the CA and may mimic reproductive dormancy.

### The CC Mediates Metabolic Homeostasis.

The CC helps maintain metabolic homeostasis throughout the animal by controlling production of its glucagon-like Akh signal (61). Akh also shows homology to mammalian gonadotrophin-releasing hormone GnRH (62), and the Akh receptor AkhR shows evolutionary conservation to the mammalian GnRH receptor (22, 63). These similarities underscore that Drosophila has tissues analogous to the vertebrate HPG (hypothalamus pituitary gonadal) axis and that the regulation of metabolism in animals has a common evolutionary origin. Our experiments greatly expand knowledge of the transcriptome of the adult CC and identify at least 39 GFP reporter-lines and Gal4 lines from the CRIMIC and MiMIC-derived collections for CC-preferential genes (*SI Appendix*, SI Dataset, Table S5).

During development, the CC detects glucose levels through changes in conserved ATP-sensitive potassium channels (61) and down-regulates Akh production in response to nutrient stress using a conserved cGMP-dependent protein kinase encoded by *dg2* (22). Production of allatostatin A (AstA) by the intestine was recently demonstrated to increase in response to sleep deprivation (64). Specifically, high AstA levels stimulate Akh release from the CC in response to AstA-R receptor activation, giving rise to energy wasting.

### The Foregut May be a Focus of Evolutionary Adaptation.

Recently, sequencing of historical Drosophila specimens from museum collections spanning the last 200 y made it possible to identify genes likely to have been targets of recent selection (65). These results supported previous evidence that selection at the *ref*(2)*P* and *CHKov1* genes contributed to sigma virus resistance beginning around 1,800. More recently, selective changes identified at *Cyp6g1* and *Ahcy* are consistent with postulated roles in DTT resistance. These results take on further interest in light of our findings showing that all four of these genes are preferentially expressed in the foregut in cluster N1 or in N1 and the CA. Genetic changes in foregut genes in response to environmental changes involving pathogen and toxin exposure are consistent with the foregut’s gatekeeper role.

### The Dynamic Nature of the PM.

This study expands upon previous analyses of the PM in diverse insects (6, 9, 11, 20, 29, 38). We clarified the likely origin of the four PM layers from different PV cell types and determined genes expressed preferentially in each to gain insight into the digestive strategy of the PM (Fig. 4*E*). Such a layered structure appears to be conserved at least over intermediate evolutionary time frames, since the PM zones as revealed by electron microscopy in *C. erythrocephala* (20) appeared very similar to our observations in Drosophila. Previous comparisons between groups have emphasized the major differences in PM types between insect groups epitomized by the classification into type I and type II PMs, which are either produced continuously along the midgut (type I) or extruded from the PV (type II) (6). While differences are inevitable given the diversity of insect lifestyles, our results suggest that the PM is a dynamic structure that changes substantially in response to dietary changes and circadian inputs. This study provides genetic tools to undertake detailed investigations of PM biology and dynamics.

Our data and previous analyses of midgut gene expression (30, 31) strongly suggest that the PM continues to be modified and supplemented with new chitin, chitin-binding proteins, digestive enzymes, and immune proteins as food materials move through the midgut. Supporting this conclusion, the same chitin synthase, Chs2, that builds the PM scaffold in the anterior PV continues to be expressed by midgut enterocytes in some regions. New chitin fibers may be synthesized near the site where they are needed, including by intestinal microvilli. Chs2 is likely activated on site by chymotrypsin-dependent proteolysis. PV6-3 cells and enterocytes throughout the midgut express high levels of *Jon99Cii* and *Jon99Ciii* which are orthologs of human chymotrypsin C (*CTRC*). *CTRC* is expressed in the pancreas as an inactive proenzyme that is activated by trypsin. It is required for full activation of other digestive proteases and degrades excess trypsinogen to protect against excess trypsin activity (66). Jon99C proteins likely activate Chs2 in a similar manner. Studies in *Manduca sexta* indicate that Chs2 is activated after its extracellular carboxy terminal region binds a chymotrypsin-like enzyme. Insect chymotrypsins are themselves activated by midgut trypsins (67). Many digestive enzymes have the structure of zymogens that can likely be secreted, assembled in the PM, and then activated by the digestive activity of the PM lumen. The anterior midgut region may be particularly active in PM remodeling. Dozens of PM-related genes and enzymes are expressed highly in the most anterior midgut region, region a1, but at much lower levels in the middle and posterior midgut (30).

### Evolution of a Protective Layer Separating the Gut Microbiome and Enterocytes.

Currently, the PM is widely viewed as an invertebrate-specific structure that functions in both digestion and to protect enterocytes from excessively activating the immune system due to direct contact with bacteria. In mammals, such protection is provided by Muc2-type mucins that form a thick protective layer whose disruption is associated with inflammatory bowel disease [review: (68)]. Our detailed analysis of the PM now suggests that these differences may be smaller than previously supposed. The large L3 layer of the PM is highly enriched in mucins and lies close to enterocytes (Fig. 4*E*). This layer is produced by PV5 cells, which are larger and more numerous than other PM-producing cells combined. It may be that a mucin layer represents the evolutionarily most ancient mechanism for protecting gut cells from the gut contents. The evolution of the outer layers and reinforcement with chitin fibrils may have evolved later to provide greater protection from mechanical damage caused by food items. We observed a possible remnant of this ancient connection in our studies of PV5 cell morphology. These distinctively shaped columnar cells contain apical secretory microvilli and lateral plasma membrane extensions that anchor PV5 cells to their neighbors (Fig. 1*B* and *SI Appendix*, Fig. S2*A*). Similar cell morphology, secretory microvilli, and lateral extensions are present in major mucus-producing goblet and adjacent Tuft cells in mammals (69, 70).

### The Foregut and Immunity.

The Drosophila gut (71), the PM (11), and associated gut microbiota (5) all make important contributions to defense against pathogens. Our observations add to the knowledge of immune gene expression in the foregut in animals growing on a normal laboratory diet low in pathogens. In this state, the esophagus and PV still express a wide range of genes with documented functions in virus and pathogen resistance. Moreover, they express sensor molecules such as *PGRP-LB* in PV6-1 cells and *PGRP-SC2* in PV6-3 cells, a few antibacterial effectors such as lysozyme genes, and antiviral *vir-1* in PV and esophageal cells. Nonetheless, the highly inducible effector molecules such as antimicrobial peptides were not highly expressed under the conditions of scRNAseq analyses.

Within the posterior PV, we detected potential sources of host immunity. We found that PV6-3 cells express several antibacterial genes including the lysozyme *LysE*, a regulator of the humoral response *Pebp1*, and the antivirus effector *CG11671*. PV6-4 cells, a much smaller cell group than PV6-2 and PV6-3, differentially expresses several genes with antimicrobial functions, including the antifungal *Drs* and the putative bacterial lysozyme *LysP*. These immune effectors also may have a role in digestion of consumed microbes to liberate their nutrients for absorption by the fly (72). PV6-4 also highly expressed a group of genes with unknown functions, including *CG45080*, *CG34212*, and *CG13947* (*SI Appendix*, SI Dataset, Tables S3 and S4). We also captured a separate small cluster of Hemolectin (*Hml*)-expressing cells. *Hml* encodes a large multidomain protein produced by hemocytes, a primary Drosophila immune cell type. To localize these cells, we expressed GFP under the control of an *Hml* Gal4 line. This approach allowed us to consistently localize the *Hml*-expressing-cells to the PV6 and anterior midgut junction (*SI Appendix*, Fig. S3*D*). Based on their posterior location, some of these immune reactive cells may account for the regionalized production of antimicrobial peptides observed in response to IMD pathway activation (73).

The esophagus (and crop duct) within the PV constitutes a foregut commensal niche that hosts strains of *L. plantarum* (*LpWF*) isolated from wild-caught *D. melanogaster* [(4); Fig. 5*A*]. The establishment of *LpWF* within the niche facilitates subsequent colonization at closely associated esophageal sites by *Acetobacter indonesiensis* (*Ai*). These microorganisms have commensal functions including nutrition, immune regulation, and protection from infection (3, 5, 74). The presence of putative commensal interactions within the esophagus, a tissue that also must protect against pathogenic bacteria and viruses within the food, suggests that the PV has mechanisms for differentiating between commensal and pathogenic microbes.

The normal Drosophila microbiome appears to actively instruct the foregut to adapt its expression of immune genes in proventricular cells to the relatively low levels we observed in our scRNAseq experiments with flies raised on normal laboratory conditions. When we raised flies axenically and then exposed them to a conventional microbiome condition during adulthood, analysis of PV transcriptomes by RNAseq showed an upregulation of antimicrobial peptides and many other immune molecules (*SI Appendix*, SI Dataset, Table S7). This response may reflect a failure to desensitize PV immunity to the presence of commensal species present at a basal level. How the foregut and downstream intestinal tissue are prepared to protect the intestine from pathogens whenever an infection occurs while selectively maintaining the commensals remains to be fully explored (5).

### Advancing scRNAseq Studies Using Experimentally Tailored High-Content Biological Annotation.

Our studies highlight a powerful way to further improve scRNAseq pipelines. Often, cell clusters can neither be fully identified nor tested for cell-type homogeneity. We employed a robust and expandable system of biological annotation by selecting more than 150 strains from a genome-wide library of Drosophila stocks carrying gene expression reporters for more than 1,800 different genes. Studying the spatial expression of these genes in the foregut tissue of flies living under similar conditions to those experienced prior to scRNAseq cell preparation minimizes many variables. Moreover, these lines provide a permanent resource for addressing future questions.

The most straightforward use of the lines is to identify and validate cell clusters. Typically, multiple genes enriched in a cluster can be tested, allowing gene expression homogeneity within the cluster to be investigated. Full specificity is not essential to gain substantial benefits in interpreting gene knockdown experiments. Further diversifying gene coverage will only increase opportunities to enhance gene expression-based studies using tissue-level biological content.

### Understanding Evolutionarily Conserved Aspects of Foregut Biology.

How tissue systems in early animals developed neuroendocrine controls to optimize the activity of physiological systems such as digestion has the potential to illuminate current animal biology. Hartenstein (13) has reviewed the striking similarity in neuroendocrine control mechanisms between phyla, but differences are also found (14). Evidence exists for the conservation of many intestinal peptide-signaling systems (75). For example, oxytocin regulation has been conserved over 600 million years (76). Segrist and Cherry (77) documented conservation of intestinal epithelial defenses against viruses. Body temperature regulation is mediated by Dh31/Dh31-R in Drosophila. In vertebrates, the Dh31-R ortholog, the calcitonin receptor (CalcR), controls body temperature (78, 79). Circadian clock regulation of many aspects of animal physiology is also conserved (80, 81). The neuroendocrine control of metabolism via the vertebrate HPE axis may extend to most or all animal groups (22). Given the many critical roles played by the foregut, further study of this tissue is certain to deepen our understanding of animal biology.

## Materials and Methods

We describe in the Results section the methods used to carry out this study using widely known techniques [(34, 36–38, 49) and *SI Appendix*, *References*]. Our use of hand-isolated, highly pure foregut starting material for cell isolation and our validation and refinement of initial clustering using biological annotation were essential to achieving high-quality results. Detailed protocols and materials information are given in *SI Appendix*, *Supplemental Methods*.

## Supplementary Material

Appendix 01 (PDF)

Dataset S1 (XLSX)

Dataset S2 (XLSX)

Dataset S3 (XLSX)

Dataset S4 (XLSX)

Dataset S5 (XLSX)

Dataset S6 (XLSX)

Dataset S7 (XLSX)

Dataset S8 (XLSX)

Movie S1. Movements of a freshly dissected proventriculus cultured *in vitro*.

## Data Availability

Sequence data have been deposited in NIH GEO (GSE243037) (37). All other data are included in the manuscript and/or supporting information.
