# Supplementary material for: Cellular and molecular organization of the Drosophila foregut
Source: Proc Natl Acad Sci U S A. 2024 Mar 5;121(11):e2318760121. doi: 10.1073/pnas.2318760121 (PMC10945768; doi:10.1073/pnas.2318760121)
Supplement: Supplementary file 1 — Appendix 01 (PDF) [file pnas.2318760121.sapp.pdf]

PNAS

Supporting Information for

Cellular and molecular organization of the Drosophila foregut

Haolong Zhu, William B. Ludington, and Allan C. Spradling

Corresponding authors: Allan C. Spradling <spradling@carnegiescience.edu>, and William B. Ludington <ludington@carnegiescience.edu>

This PDF file includes:

Supplemental materials and methods
Figures S1 to S4
Legends for Datasets S1 to S8
Legend for Movie S1
SI References

Other supporting materials for this manuscript include the following:

Datasets S1 to S8
Movie S1

Supplemental materials and methods

Drosophila stocks, husbandry, and crosses

Drosophila were cultured on the standard laboratory fly food at room temperature (23 to 25 °C). Genes driving Gal4 were examined after crossing to UAS-GFP reporters. Genes fused to GFP were examined directly. Drosophila stocks used in the study are summarized in the key resources table.

Immunofluorescence and light microscopy

Drosophila guts were dissected in phosphate-buffered saline (PBS) followed by immediate fixation in 4% paraformaldehyde in PBS (made from Paraformaldehyde 16% Aqueous Solution EM Grade, Electron Microscopy Sciences, Sku#15710) for 90 minutes at room temperature. Samples were then washed and permeabilized three times in PBST (0.1% Triton X-100 in PBS) for 15 minutes each. Samples were stained with DAPI (2 µg/mL in PBST) for 10 minutes at room temperature, and then washed three times in PBST for 15 minutes each. Samples were equilibrated in 50% glycerol in PBST for at least 30 minutes, and then mounted in mounting media (4% propyl gallate, Sigma-Aldrich, Sku#02370, and 80% glycerol in PBST). All the incubation and washing steps were performed on a nutator. Images were acquired with a Leica TCS SP8 confocal microscope or a Leica Stellaris 8 DIVE multiphoton confocal.

IMARIS nucleus quantification

Proventriculus cell quantification was performed using the spot detection function of the IMARIS software (v10.0.0). Specifically, the raw Z-stack data was reconstructed into a 3D object from which spots were generated by automated spot detection based on

the DAPI signal. Cell type boundaries were determined based on the cellular identities and later verified by transgenic labeling. The results were validated manually and multiplets were removed when necessary.

***Ex vivo* live imaging**

Ex vivo foregut live imaging was conducted by following Marchetti, Zhang, and Edgar (2022) with minor modifications as noted. Briefly, Drosophila gut tissues were freshly dissected and mounted in agarose pads made of 1% low gelling temperature agarose (Sigma-Aldrich, Sku#A9414) in modified Schneider's medium without additives. Mounted samples were placed in a 35 mm dish (ibidi, µ-Dish 35 mm low, Cat#80136). About 120 µL of the complete culture medium with no isradipine was carefully added to the dish. See Marchetti, Zhang, and Edgar (2022) for the detailed recipe. Live imaging was performed on a Leica DMI8 inverted microscope equipped with the Hamamatsu Flash4 v3 sCMOS camera. Images were acquired using the DIC mode at 100 fps. The movie was generated from the raw data using the VisiView software (Visitron Systems, v6.0.0.18), IMARIS (v10.0.0), and Adobe Premiere (v22.64).

RNA fluorescence *in situ* hybridization (RNA-FISH)

RNA-FISH probes were designed using the Stellaris Probe designer (https://www.biosearchtech.com/support/tools/design-software/stellaris-probe-designer) and synthesized with the Stellaris Probe Technology (Biosearch Technology Inc.). The probe sequences are summarized in Dataset S8. RNA-FISH experiments were performed by following the Stellaris RNA-FISH *D. melanogaster* Wing Imaginal Discs protocol (Biosearch Technology Inc.). Briefly, dissected proventriculus tissues were fixed in 4% PFA in PBS and permeabilized in 70% ethanol in PBS overnight at 4 °C. Samples were

washed in 1 mL of Wash Buffer A (Cat#SMF-WA1-60) for 5 minutes at room temperature, and then incubated in 100 µL of Hybridization Buffer (Cat#SMF-HB1-10) containing probe (1 µL of 5 nmol probe stock solution to 100 µL of Hybridization Buffer) for 10-16 hours at 37 °C. Wash Buffer A and Hybridization Buffer contain 10% (v/v) formamide. Samples were washed in 1mL of Wash Buffer A for 30 minutes at 37 °C followed by DAPI nuclear stain (2 µg/mL DAPI in Wash Buffer A) for 30 minutes at 37 °C. Afterward, samples were washed in 1 mL of Wash Buffer B (Cat#SMF-WB1-20) for 5 minutes at room temperature and finally mounted in mounting media (4% propyl gallate, Sigma-Aldrich, Sku#02370 and 80% glycerol in PBST). All the steps were performed on a nutator. Images were acquired with a Leica TCS SP8 confocal microscope.

Electron microscopy

Samples for electron microscopy were prepared as described in Dodge *et al.* 2023 with minor modifications as noted. Drosophila foreguts were dissected and fixed in 1% paraformaldehyde (made from Paraformaldehyde 16% Aqueous Solution EM Grade, Electron Microscopy Sciences, Sku#15710), 3% glutaraldehyde (made from Aqueous Glutaraldehyde EM Grade 25%, Electron Microscopy Sciences, Sku#16220), 2 mM Calcium Chloride, and 0.1 M Cacodylate buffer (pH 7.4) for one hour at room temperature and then overnight at 4 °C. After fixation, samples were quenched in 50 mM Glycine, 0.1 M Cacodylate buffer for 15 minutes, washed with 0.1 M Cacodylate buffer 2 times, and post-fixed with 1% osmium tetroxide (made from Osmium Tetroxide 4% Aqueous Solution, Electron Microscopy Sciences, Sku#19150) and 1.5% potassium ferrocyanide in 0.1 M Cacodylate buffer for 1 hour at room temperature. Samples were

washed with Cacodylate buffer and then 0.05 M Maleate buffer (pH 5.15) followed by *en bloc* staining with 0.5% (w/v) uranyl acetate (Electron Microscopy Sciences, Cat#22400) in 0.05 M Maleate buffer (pH 5.15) for 1 hour. After washing in water 3 times, samples were dehydrated using 30%, 50%, 75%, 85%, 95%, and 100% ethanol in series, followed by four rinses in propylene oxide. After dehydration, samples were infiltrated and embedded in EMbed 812 resin (Electron Microscopy Sciences, Sku# 14900) following the manufacturer's protocols. The resin blocks were oriented and cross-sectioned using a Leica EMUC7 ultramicrotome. Ultrathin sections at about 85 nm thickness were collected at every 25-30-micron interval and examined using a Hitachi HT7800 transmission electron microscope operated at 80KeV. Digital images were acquired by using an AMT NanoSprint 12 camera.

Single cell RNA sequencing (scRNAseq)

Proventricular and associated tissues were dissected in cold PBS from adult mated female flies (w[1118]; P{w[+mC]=UAS-GFP.nls}/+; PBac{w[+mC]=IT.GAL4}bond[1385-G4]/+) reared with a conventional gut microbiome. Tissue-cutting sites were at the anterior esophagus before its entry point into the proventriculus, the anterior midgut boundary, and the crop duct entering the base of the crop (Figure S1A). Dissected tissues were kept in PBS on ice until about 60-70 pieces were collected. Each proventriculus was delaminated from a triple-layer epithelium into a monolayer by gentle tugging with forceps before further dissociation (Figure S1B). The tissue suspension was processed with a combined mechanical and enzymatic dissociation (8-10 mg/mL Gibco Collagenase, Type IV, powder, Thermo Fisher, Cat#17104019 in PBS) for about 30 minutes at room temperature. After the dissociation, 150 µL FBS was

added to quench the reaction. The cell suspension was filtered through a 20 µm syringe (BD, Cat#340595). The cells were spun down at 500 g for 5 minutes at 4℃ following supernatant removal. The cell pellets were washed in 1 mL of cold PBS with 0.08% BSA with a gentle resuspending. The cells were spun down again at 500 g for 5 minutes at 4℃ and resuspended in 40 µL PBS with 0.08% BSA. A small portion of the cell suspension was examined on a hemocytometer from which single cells with various sizes and morphologies were observed. Libraries were prepared using 10X Genomics products including the Chromium Next GEM Single Cell 3' Kit v3.1 (PN1000128), Chromium Next GEM Chip G Single Cell Kit (PN1000127), and Single Index Kit T Set A (PN1000213) and following the manufacturer's protocol (CG000204 Rev D). Qubit dsDNA Quantification Assay Kit (Thermo Fisher, Cat#Q32850) and the Agilent High Sensitivity DNA Kit (Agilent, Cat#5067-4626) on the 2100 Bioanalyzer System were used at the appropriate steps for quantification and quality control. Sequencing was conducted on the Illumina NextSeq500 using 28x91 bp reads with 8 base indexing, as per the 10X Genomics protocol.

Analyses of scRNAseq

Reads were processed using Cell Ranger (v6.0.1) following the 10X Genomics protocol. The *Drosophila melanogaster* genome BDGP6.22 Ensemble release 98 was used as the reference genome. A piece of *GFP* CDS sequence (Dataset S7) was added as an artificial chromosome in the reference genome to allow *GFP* expression detection. Further bioinformatic analyses were performed using the Seurat package (v4.3.0) following the published instructions from the Satija lab in R (v4.1.2). Putative cells with a high ratio of mitochondrial gene expression (mtRNA > 8%) or/and abnormally high

UMI (UMI > 5000) were eliminated. Triplicate datasets were integrated using the Seurat integration function. The initial clustering identified 18 putative cell clusters (Fig 2A). One initial cluster (#2) with an abnormally higher average UMI than the others and mixed molecular identities was removed. A comprehensive re-clustering was conducted to further improve the signal sensitivity by examining the analysis with different numbers of principal components and the resolution parameter (PCs = 30, resolution = 0.3). Sub-clustering was introduced to distinguish a few clusters containing multiple cell types. These steps were essential to identifying the balance of over- and under-clustering and maximizing the detection of true biological signals. Overall, the analyses determined 18 final cell clusters which were further validated by transcriptomic analyses and genetic labelling. Plots were generated using the ggplot2 package (v 2_3.4.3).

Raising axenic flies

Axenic flies were raised by following Dodge *et al.* (2023). Briefly, germ-free Canton-S flies were reared in Wide Drosophila Vials (Cat#32-114, Genesee), with Droso-Plugs (Cat#59-201, Genesee). The germ-free fly food composition was 10% glucose (filter-sterilized), 5% autoclaved live yeast, 0.42% propionic acid (filter-sterilized), 1.2% autoclaved agar, and 0.5% autoclaved cornmeal. Each vial contained about 3-5 mL of food. Germ-free fly stocks were passaged to fresh vials every 3-4 days.

Bulk messenger RNA sequencing (RNAseq)

Flies used for RNAseq were reared axenically. Flies except the axenic proventriculus control samples were inoculated with a dose of *Lactiplantibacillus plantarum* (*LpWF*, Obadia *et al.* 2017 and Dodge *et al.* 2023), one of the *Drosophila melanogaster* core gut microbiome species, during adulthood. For *LpWF* inoculation

(Obadia *et al.* 2017 and Dodge *et al.* 2023), an equivalent of 10^5-10^6 CFU/fly *LpWF* cell suspension in PBS was spread on the top of the fly food where adult flies were housed.

For the proventriculus isolation, about 30 pieces of the proventriculus tissue (with minimum tissues from crop ducts) from adult mated female flies were dissected in cold PBS. For crop isolation, about 30 pieces of the crop tissue (including crop ducts) from adult mated female flies were dissected in cold PBS. Excess PBS was removed as much as possible from the tissue collection after a quick spin-down. Samples were flash frozen in liquid nitrogen and stored at -80 °C until enough samples were collected. RNA extractions were performed using the Qiagen RNeasy Mini Kit (50) (Qiagen, Cat#74104) and Qiagen RNase-Free DNase Set (50) (Qiagen, Cat# 79254) and following the manufacturer's protocols.

For the salivary gland isolation, about 20 pairs of salivary gland tissues from adult mated female flies were dissected in cold PBS. Excessive PBS was removed as much as possible from the tissue collection after a quick spin-down. Samples were resuspended in an appropriate amount of TRIzol Reagent (Thermo Fisher, Cat#15596026) following the manufacturer's protocols. Samples were flash frozen in liquid nitrogen and stored at -80 °C until enough samples were collected. RNA extractions were performed using the TRIzol Reagent (Thermo Fisher, Cat#15596026) and following the manufacturer's protocols.

For all RNAseq experiments, purified total RNA samples were quantified and examined using the Qubit RNA High Sensitivity (HS) Assay Kit (Thermo Fisher, Cat#Q32852) and the Agilent RNA 6000 Pico Kit (Agilent, Cat#5067-1513) on the 2100

Bioanalyzer System for the quantification and quality control. The TruSeq Stranded Total RNA Library Prep Human/Mouse/Rat, 48 Samples kit (Illumina, Cat#20020596) was used for proventriculus and crop samples, and the TruSeq Stranded mRNA Library Prep, 48 Samples kit (Illumina, Cat#20020594) was used for salivary gland samples, following the manufacturer's protocols. The indexes used were from TruSeq RNA Single Indexes Set A, 12 Indexes, 48 Samples kit (Illumina, Cat#20020492). Sequencing was conducted on the Illumina NextSeq500 using 75bp single-end reads.

Analyses of RNAseq

RNAseq data was processed using the Nextflow (v22.10.1) workflow revision 3.11.1. The *Drosophila melanogaster* genome BDGP6.22 Ensemble release 98 was used as the reference genome. For differential expression analysis on the proventriculus samples, Salmon-package-generated gene count data were further analyzed with the DESeq2 package (v1.34.0) in R (v4.1.2). Plots were generated using the ggplot2 package (v2_3.4.3).

Generation of transgenic flies

The Muc68D-tag line was generated using the plasmid containing the Muc68D-tag construct (ConstructID#35649249831002394_H07) generated by Sarov *et al.* (2016). The plasmid was injected into the embryos of the recipient fly line with germline phiC31expression and the attP40 landing site (yw, nos-PhiC31; attP40). Flies with successful recombination events as indicated by the dsRed phenotypic marker were selected and balanced.

The vir-1-GFP line was generated based on the MiMIC line MI04501 whose insertion was located in the first *vir-1* coding intronic region by following the MiMIC RMCE protocol (Li-Kroeger *et al.* 2018).

Reanalysis of esophagus X-ray micro-computed tomography (XR µCT) data

The X-ray micro-computed tomography (XR µCT) data for the adult fly esophagus from Dodge *et al.* (2023) was reanalyzed using IMARIS software (v10.0.0). Specifically, samples with *LpWF* colonization, and *LpWF* and *Acetobacter indonesiensis* (*Ai*) co-colonization were examined. Samples were reoriented and resampled with the esophagus perpendicular to the plane of the screen using the free rotation function. Relative esophageal space along the anterior-pastier axis was quantified and plotted using the surface function.

Key resources table

**Reagent or Resource**	**Source**	**Identifier**
Bacterial and virus strains		
Lactobacillus plantarum	W. B. Ludington	Obadia *et al.* (2017) & Dodge *et al.* (2023)
Chemicals, peptides, and recombinant proteins		
Paraformaldehyde 16% Aqueous Solution EM Grade	Electron Microscopy Sciences	Sku#15710
Propyl gallate	Sigma-Aldrich	Sku#02370
Low gelling temperature agarose	Sigma-Aldrich	Sku#A9414
Wash Buffer A	Biosearch Technology Inc.	Cat#SMF-WA1-60
Wash Buffer B	Biosearch Technology Inc.	Cat#SMF-WB1-20
Hybridization Buffer	Biosearch Technology Inc.	Cat#SMF-HB1-10
EMbed 812 resin	Electron Microscopy Sciences	Sku# 14900

Aqueous Glutaraldehyde EM Grade 25%	Electron Microscopy Sciences	Sku#16220
Osmium Tetroxide 4% Aqueous Solution	Electron Microscopy Sciences	Sku#19150
Uranyl acetate	Electron Microscopy Sciences	Cat#22400
Gibco Collagenase, Type IV, powder	Thermo Fisher	Cat#17104019
TRIzol Reagent	Thermo Fisher	Cat#15596026
Critical commercial assays		
Chromium Next GEM Single Cell 3’ Kit v3.1	10X Genomics	PN1000128
Chromium Next GEM Chip G Single Cell Kit	10X Genomics	PN1000127
Single Index Kit T Set A	10X Genomics	PN1000213
Qubit dsDNA Quantification Assay Kit	Thermo Fisher	Cat#Q32850
Qubit RNA High Sensitivity (HS) Assay Kit	Thermo Fisher	Cat#Q32852
High Sensitivity DNA Kit	Agilent	Cat#5067-4626
RNA 6000 Pico Kit	Agilent	Cat#5067-1513
RNeasy Mini Kit (50)	Qiagen	Cat#74104
RNase-Free DNase Set (50)	Qiagen	Cat# 79254
TruSeq Stranded Total RNA Library Prep Human/Mouse/Rat, 48 Samples kit	Illumina	Cat#20020596
TruSeq Stranded mRNA Library Prep, 48 Samples kit	Illumina	Cat#20020594
TruSeq RNA Single Indexes Set A, 12 Indexes, 48 Samples kit	Illumina	Cat#20020492
Deposited data		
Drosophila foregut (PV and associated tissue) scRNA-seq	GEO	GSE243037
Drosophila PV bulk RNA-seq	GEO	GSE243037
Drosophila Crop bulk RNA-seq	GEO	GSE243037
Drosophila SG bulk RNA-seq	GEO	GSE243037
Experimental models: Organisms/strains		
w[1118]; P{w[+mC]=UAS-GFP.nls}14	BDSC	4775
y[1] w[*]; P{w[+mC]=UAS-mCD8::GFP.L}LL5, P{UAS-mCD8::GFP.L}2	BDSC	5137
w[*]; P{w[+mC]=UASp-GFPS65C-alphaTub84B}3/TM3, Sb[1]	BDSC	7373
Canton-S	W. B. Ludington	CSWF
w[1118]	BDSC	3605
w[*]; P{GAL4E69}puc[GAL4E69]/TM3, Sb[1]	BDSC	6762
w[1118]; P{w[+mW.hs]=GawB}path[c135]	BDSC	6978

w[1118]; P{w[+mC]=drm-GAL4.7.1}1.1/TM3, Sb[1]	BDSC	7098
w[1118]; P{w[+mGT]=GT1}nrv3[BG01034]	BDSC	12761
y[1] w[67c23]; Mi{GFP[E.3xP3]=ET1}cpx[MB00267]	BDSC	22750
y[1] w[67c23]; Mi{GFP[E.3xP3]=ET1}AstC-R2[MB01754]	BDSC	23355
w[1118]; Mi{GFP[E.3xP3]=ET1}CG14830[MB02495]	BDSC	23446
w[1118]; Mi{GFP[E.3xP3]=ET1}Jhbp12[MB02652]	BDSC	23455
w[1118]; Mi{GFP[E.3xP3]=ET1}sprt[MB02257]	BDSC	23795
y[1] w[67c23]; Mi{GFP[E.3xP3]=ET1}CG8180[MB01013]	BDSC	23953
w[1118]; Mi{GFP[E.3xP3]=ET1}Jhbp14[MB04780]	BDSC	24226
w[1118]; Mi{GFP[E.3xP3]=ET1}Cpr51A[MB03837]	BDSC	24278
w[1118] Mi{GFP[E.3xP3]=ET1}CG15043[MB04204]	BDSC	24573
w[1118] Mi{GFP[E.3xP3]=ET1}CG32694[MB05037]	BDSC	24601
w[1118]; Mi{GFP[E.3xP3]=ET1}Npc2b[MB04347]	BDSC	24694
w[1118]; Mi{GFP[E.3xP3]=ET1}CG14125[MB04957]	BDSC	24731
w[1118]; Mi{GFP[E.3xP3]=ET1}Pgant4[MB04930]/SM6a	BDSC	24790
w[1118]; Mi{GFP[E.3xP3]=ET1}cpx[MB05709]	BDSC	25231
w[1118]; Mi{GFP[E.3xP3]=ET1}CG15236[MB07522] CG34215[MB07522]	BDSC	25556
w[1118]; Mi{GFP[E.3xP3]=ET1}Amy-d[MB09212]	BDSC	26451
w[1118]; Mi{GFP[E.3xP3]=ET1}CG34045[MB11449]	BDSC	27867
w[1118]; Mi{GFP[E.3xP3]=ET1}CG1299[MB09811]	BDSC	29063
w[*]; P{w[+mW.hs]=GawB}Aug21/CyO	BDSC	30137
w[1118]; P{y[+t7.7] w[+mC]=GMR54F05-GAL4}attP2	BDSC	39080

w[1118]; P{y[+t7.7] w[+mC]=GMR58E05-GAL4}attP2	BDSC	39182
w[1118]; P{y[+t7.7] w[+mC]=GMR58H06-GAL4}attP2	BDSC	39199
w[1118]; P{y[+t7.7] w[+mC]=GMR66A07-GAL4}attP2	BDSC	39385
y[1] w[*]; Mi{y[+mDint2]=MIC}vir-1[MI04501]	BDSC	40201
w[1118]; P{y[+t7.7] w[+mC]=GMR66A12-GAL4}attP2	BDSC	41296
w[1118]; PBac{y[+mDint2] w[+mC]=Dif-GFP.FPTB}VK00033	BDSC	42673
w[1118]; P{y[+t7.7] w[+mC]=GMR12B10-GAL4}attP2	BDSC	48490
w[1118]; P{y[+t7.7] w[+mC]=GMR12C02-GAL4}attP2	BDSC	48493
w[1118]; P{y[+t7.7] w[+mC]=GMR12D09-GAL4}attP2	BDSC	48503
w[1118]; P{y[+t7.7] w[+mC]=GMR13A04-GAL4}attP2	BDSC	48537
w[1118]; P{y[+t7.7] w[+mC]=GMR13B01-GAL4}attP2	BDSC	48541
w[1118]; P{y[+t7.7] w[+mC]=GMR13D09-GAL4}attP2	BDSC	48561
w[1118]; P{y[+t7.7] w[+mC]=GMR13D11-GAL4}attP2	BDSC	48562
w[1118]; P{y[+t7.7] w[+mC]=GMR13F11-GAL4}attP2	BDSC	48579
w[1118]; P{y[+t7.7] w[+mC]=GMR13G01-GAL4}attP2	BDSC	48580
w[1118]; P{y[+t7.7] w[+mC]=GMR66A03-GAL4}attP2	BDSC	49615
w[*]; P{w[+mC]=PTT-GC}Tm1[CC00578]/TM3, Ser[1] Sb[1]	BDSC	51537
y[1] w[*]; PBac{y[+mDint2] w[+mC]=repo-GFP.FPTB}VK00037/CyO	BDSC	56153
y[1] w[*] Mi{PT-GFSTF.1}rst[MI04842-GFSTF.1] kirre[MI04842-GFSTF.1]/FM7j, Bar[1]	BDSC	59410
y[1] w[*]; Mi{PT-GFSTF.1}nkd[MI00209-GFSTF.1]/TM3, Sb[1] Ser[1]	BDSC	59759
y[1] w[*]; Mi{PT-GFSTF.1}Eip63E[MI00413-GFSTF.1]/TM6C, Sb[1] Tb[1]	BDSC	59763
y[1] w[*]; Mi{PT-GFSTF.0}Dys[MI01893-GFSTF.0]/TM3, Sb[1] Ser[1]	BDSC	59782

y[1] w[67c23]; Mi{PT-GFSTF.0}Syt1[MI02197-GFSTF.0]/CyO	BDSC	59788
y[1] w[*]; Mi{PT-GFSTF.0}Rdl[MI02620-GFSTF.0]/TM6C, Sb[1] Tb[1]	BDSC	59796
y[1] w[*]; Mi{PT-GFSTF.2}Jupiter[MI00919-GFSTF.2]	BDSC	60156
y[1] w[67c23]; Mi{PT-GFSTF.1}meng[MI03008-GFSTF.1] CG44271[MI03008-GFSTF.1-X]	BDSC	60191
y[1] w[*]; Mi{PT-GFSTF.0}CG10226[MI04110-GFSTF.0]/TM6C, Sb[1] Tb[1]	BDSC	60208
y[1] w[*] Mi{PT-GFSTF.0}Dop2R[MI09401-GFSTF.0]	BDSC	60276
y[1] w[67c23]; Mi{PT-GFSTF.1}Dif[MI05295-GFSTF.1]/CyO	BDSC	60506
y[1] w[*]; Mi{PT-GFSTF.1}tok[MI06118-GFSTF.1]/TM6C, Sb[1] Tb[1]	BDSC	60550
y[1] w[67c23]; Mi{PT-GFSTF.1}robo2[MI04295-GFSTF.1]	BDSC	61774
y[1] w[67c23]; Mi{PT-GFSTF.1}CG34045[MI08022-GFSTF.1] Rgk1[MI08022-GFSTF.1-X]	BDSC	61798
w[1118]; PBac{w[+mC]=IT.GAL4}Ae2[0056-G4]	BDSC	62603
w[1118]; PBac{w[+mC]=IT.GAL4}CG14830[0131-G4]	BDSC	62655
w[1118]; PBac{w[+mC]=IT.GAL4}galectin[0154-G4] asRNA:CR44987[0154-G4]	BDSC	62672
w[1118] PBac{w[+mC]=IT.GAL4}Evi5[0291-G4]	BDSC	62760
y[1] w[*]; Mi{PT-GFSTF.2}mfas[MI11275-GFSTF.2]	BDSC	63204
w[1118]; PBac{w[+mC]=IT.GAL4}0456-G4	BDSC	63331
w[1118]; PBac{w[+mC]=IT.GAL4}0495-G4	BDSC	63358
w[1118]; PBac{w[+mC]=IT.GAL4}galectin[0580-G4] asRNA:CR44987[0580-G4]	BDSC	63422
w[1118]; PBac{w[+mC]=IT.GAL4}Atpalpha[0712-G4]/TM6B, Tb[1]	BDSC	63486
w[1118]; PBac{w[+mC]=IT.GAL4}puc[0769-G4]/TM6B, Tb[1]	BDSC	63509

w[1118]; PBac{w[+mC]=IT.GAL4}0959-G4/TM6B, Tb[1]	BDSC	63927
y[1] w[*]; Mi{PT-GFSTF.1}rols[MI02479-GFSTF.1]	BDSC	64471
w[1118]; PBac{w[+mC]=IT.GAL4}Atpalpha[0359-G4]/TM6B, Tb[1]	BDSC	64715
w[1118]; PBac{w[+mC]=IT.GAL4}Cipc[1261-G4]	BDSC	65524
w[1118]; PBac{w[+mC]=IT.GAL4}lbm[1252-G4]	BDSC	65640
w[1118]; PBac{w[+mC]=IT.GAL4}vir-1[1293-G4]	BDSC	65650
w[1118]; PBac{w[+mC]=IT.GAL4}bond[1385-G4]	BDSC	65697
y[1] w[*]; Mi{PT-GFSTF.2}CG14515[MI02540-GFSTF.2]	BDSC	66773
y[1] w[*]; Mi{Trojan-GAL4.1}Tsp[MI02751-TG4.1]/SM6a	BDSC	76154
y[1] w[*]; Mi{Trojan-GAL4.2}CG7997[MI05592-TG4.2]	BDSC	76174
y[1] w[*]; Mi{Trojan-GAL4.0}sprt[MI01099-TG4.0]/SM6a	BDSC	76624
y[1] w[*]; Mi{Trojan-GAL4.0}CG32444[MI08006-TG4.0]/TM3, Sb[1] Ser[1]	BDSC	76692
w[1118]; PBac{w[+mC]=IT.GAL4}Ae2[0178-G4]/TM6B, Tb[1]	BDSC	77525
y[1] w[*]; Mi{Trojan-GAL4.0}Ppn[MI03189-TG4.0]/TM3, Sb[1] Ser[1]	BDSC	77733
y[1] w[*]; Mi{Trojan-GAL4.0}qua[MI07365-TG4.0]	BDSC	77780
y[1] w[*]; Mi{Trojan-GAL4.0}Ae2[MI07482-TG4.0]/TM3, Sb[1] Ser[1]	BDSC	77781
y[1] w[*] Mi{Trojan-GAL4.2}fne[MI09399-TG4.2]	BDSC	77796
y[1] w[*]; Mi{Trojan-GAL4.0}Gfat1[MI11277-TG4.0]/TM3, Sb[1] Ser[1]	BDSC	77817
y[*] w[*] P{w[+mC]=UAS-2xEYFP}AX; Mi{Trojan-GAL4.1}cpx[MI09455-TG4.1] Nepl11[MI09455-TG4.1-X]	BDSC	77845
y[1] w[*]; TI{GFP[3xP3.cLa]=CRIMIC.GT14}ATPCL[CR00023]	BDSC	78867

y[1] w[*]; TI{GFP[3xP3.cLa]=CRIMIC.GT36}Ass[CR00189]/TM3, Sb[1] Ser[1]	BDSC	78880
y[1] w[*]; TI{GFP[3xP3.cLa]=CRIMIC.TG4.2}CG16758[CR00351-TG4.2]/TM3, Sb[1] Ser[1]	BDSC	78896
y[1] w[*]; TI{GFP[3xP3.cLa]=CRIMIC.TG4.1}Taldo[CR00404-TG4.1]	BDSC	78904
y[1] w[*] TI{GFP[3xP3.cLa]=CRIMIC.TG4.1}vanin-like[CR00524-TG4.1]/FM7h	BDSC	78938
y[1] w[*]; TI{GFP[3xP3.cLa]=CRIMIC.TG4.0}Nox[CR00693-TG4.0]/SM6a	BDSC	78988
y[1] w[*]; TI{GFP[3xP3.cLa]=CRIMIC.TG4.0}retn[CR00697-TG4.0]/SM6a	BDSC	78990
y[1] w[*]; TI{GFP[3xP3.cLa]=CRIMIC.TG4.0}Agps[CR00286-TG4.0]/SM6a	BDSC	79234
y[1] w[*]; TI{GFP[3xP3.cLa]=CRIMIC.TG4.1}Tg[CR00407-TG4.1]	BDSC	79255
y[1] w[*]; TI{GFP[3xP3.cLa]=CRIMIC.TG4.1}levy[CR00806-TG4.1]	BDSC	79295
y[1] w[*] TI{GFP[3xP3.cLa]=CRIMIC.TG4.0}CG34325[CR00600-TG4.0]	BDSC	80604
y[1] w[*]; TI{GFP[3xP3.cLa]=CRIMIC.TG4.1}CG8321[CR00803-TG4.1]/SM6a	BDSC	80673
w[1118]; P{w[+mC]=to-GAL4.D}36.9	BDSC	80938
y[1] w[*]; TI{GFP[3xP3.cLa]=CRIMIC.TG4.0}Men[CR01134-TG4.0]/TM3, Sb[1] Ser[1]	BDSC	81185
y[1] w[*]; TI{GFP[3xP3.cLa]=CRIMIC.TG4.0}Rnf146[CR00320-TG4.0]	BDSC	83169
y[1] w[*]; TI{GFP[3xP3.cLa]=CRIMIC.TG4.2}CG5938[CR00346-TG4.2]	BDSC	83171
y[1] w[*]; TI{GFP[3xP3.cLa]=CRIMIC.TG4.0}Inos[CR00890-TG4.0]/SM6a	BDSC	83197

y[1] w[*]; TI{GFP[3xP3.cLa]=CRIMIC.TG4.2}sob[CR01007-TG4.2]/SM6a	BDSC	83247
y[1] w[*]; TI{GFP[3xP3.cLa]=CRIMIC.TG4.0}sut2[CR01187-TG4.0]	BDSC	83255
y[1] w[*]; TI{GFP[3xP3.cLa]=CRIMIC.TG4.0}pio[CR01367-TG4.0]/SM6a	BDSC	83290
y[1] w[*]; TI{GFP[3xP3.cLa]=CRIMIC.TG4.0}magu[CR01196-TG4.0]/SM6a	BDSC	86339
y[1] w[*]; TI{GFP[3xP3.cLa]=CRIMIC.TG4.0}Cpr49Ae[CR01372-TG4.0]	BDSC	86347
y[1] w[*]; TI{GFP[3xP3.cLa]=CRIMIC.TG4.1}nahoda[CR01402-TG4.1]	BDSC	86362
y[1] w[*]; TI{GFP[3xP3.cLa]=CRIMIC.TG4.1}CG8180[CR01414-TG4.1]	BDSC	86370
y[1] w[*]; TI{GFP[3xP3.cLa]=CRIMIC.TG4.2}CG4267[CR01431-TG4.2]	BDSC	86382
y[1] w[*]; TI{GFP[3xP3.cLa]=CRIMIC.TG4.2}CG8306[CR01439-TG4.2]	BDSC	86388
y[1] w[*]; TI{GFP[3xP3.cLa]=CRIMIC.TG4.0}CG15120[CR01507-TG4.0]	BDSC	86425
y[1] w[*]; TI{GFP[3xP3.cLa]=CRIMIC.TG4.0}CG8785[CR01511-TG4.0]	BDSC	86427
y[1] w[*]; TI{GFP[3xP3.cLa]=CRIMIC.TG4.1}PMP34[CR01574-TG4.1]	BDSC	86452
y[1] w[*] TI{GFP[3xP3.cLa]=CRIMIC.TG4.2}Ggt-1[CR01626-TG4.2]	BDSC	86474
y[1] w[*] TI{GFP[3xP3.cLa]=CRIMIC.TG4.2}CG6048[CR01625-TG4.2]	BDSC	91267
y[1] w[*]; TI{GFP[3xP3.cLa]=CRIMIC.TG4.0}Ssk[CR01714-TG4.0] CG42674[CR01714-TG4.0-X]/TM3, Sb[1] Ser[1]	BDSC	91280

y[1] w[*]; TI{GFP[3xP3.cLa]=CRIMIC.TG4.0}Msr-110[CR01724-TG4.0]/TM3, Sb[1] Ser[1]	BDSC	91285
y[1] w[*]; TI{GFP[3xP3.cLa]=CRIMIC.TG4.2}CG8560[CR01873-TG4.2]/TM3, Sb[1] Ser[1]	BDSC	91422
y[1] w[*]; TI{GFP[3xP3.cLa]=CRIMIC.TG4.1}Jhbp12[CR02124-TG4.1]	BDSC	91442
y[1] w[*]; TI{GFP[3xP3.cLa]=CRIMIC.TG4.1}elm[CR02138-TG4.1]	BDSC	91451
y[1] w[*]; TI{GFP[3xP3.cLa]=CRIMIC.TG4.0}yellow-h[CR02253-TG4.0]	BDSC	91493
y[1] w[*]; TI{GFP[3xP3.cLa]=CRIMIC.TG4.0}Tsp42Er[CR02398-TG4.0]/SM6a	BDSC	91522
y[1] w[*]; TI{GFP[3xP3.cLa]=CRIMIC.TG4.1}CG30197[CR02408-TG4.1] Cpr51A[CR02408-TG4.1-X]/SM6a	BDSC	92254
y[1] w[*]; TI{GFP[3xP3.cLa]=CRIMIC.TG4.1}Jhbp14[CR02482-TG4.1]/SM6a	BDSC	92282
y[1] w[*]; P{y[+t7.7] w[+mC]=Drgx-GFP.FPTB}attP40	BDSC	92369
y[1] w[*]; PBac{y[+mDint2] w[+mC]=zfh1-GFP.FPTB}VK00037	BDSC	92628
y[1] w[*]; TI{GFP[3xP3.cLa]=CRIMIC.TG4.1}Idgf6[CR02424-TG4.1] Pgant9[CR02424-TG4.1-X]/SM6a	BDSC	92664
y[1] w[*]; TI{GFP[3xP3.cLa]=CRIMIC.TG4.1}CG17549[CR02452-TG4.1]/SM6a	BDSC	92671
y[1] w[*]; TI{GFP[3xP3.cLa]=CRIMIC.TG4.1}CG30371[CR02459-TG4.1]/SM6a	BDSC	92675
y[1] w[*]; TI{GFP[3xP3.cLa]=CRIMIC.TG4.1}Mfe2[CR70121-TG4.1] kat80[CR70121-TG4.1-X]/FM7h	BDSC	92712
w[*]; TI{GFP[3xP3.cLa]=CRIMIC.TG4.2}Cpr49Ae[CR60007-TG4.2]/CyO	BDSC	93295

y[1] w[*]; TI{GFP[3xP3.cLa]=CRIMIC.TG4.1}wat[CR70197-TG4.1]/TM3, Sb[1] Ser[1]	BDSC	93374
w[*]; TI{GFP[3xP3.cLa]=CRIMIC.TG4.0}CG9649[CR60129-TG4.0]/TM3, Sb[1]	BDSC	93470
y[1] w[*] TI{GFP[3xP3.cLa]=CRIMIC.TG4.1}CG3823[CR60225-TG4.1]/FM6, w[1] Myc[+]	BDSC	93542
y[1] w[*] TI{GFP[3xP3.cLa]=CRIMIC.TG4.1}CG6067[CR60226-TG4.1]/FM6, w[1] Myc[+]	BDSC	93543
y[1] w[*]; TI{GFP[3xP3.cLa]=CRIMIC.TG4.0}Vha68-2[CR70212-TG4.0]/SM6a	BDSC	93800
y[1] w[*] TI{GFP[3xP3.cLa]=CRIMIC.TG4.0}ogre[CR02576-TG4.0]/FM7h	BDSC	93923
y[1] w[*] TI{GFP[3xP3.cLa]=CRIMIC.TG4.0}CG4928[CR02584-TG4.0]	BDSC	93927
w[*] TI{GFP[3xP3.cLa]=CRIMIC.TG4.2}CG13003[CR60302-TG4.2]/FM7c	BDSC	94372
w[*]; TI{GFP[3xP3.cLa]=CRIMIC.TG4.1}CG3819[CR60333-TG4.1]/TM3, Sb[1] Ser[1]	BDSC	94380
y[1] w[*] TI{GFP[3xP3.cLa]=CRIMIC.TG4.1}spirit[CR60353-TG4.1]	BDSC	94395
w[*] TI{GFP[3xP3.cLa]=CRIMIC.TG4.1}CG15347[CR60354-TG4.1]	BDSC	94396
w[*]; TI{GFP[3xP3.cLa]=CRIMIC.TG4.1}wls[CR60365-TG4.1]/TM3, Sb[1] Ser[1]	BDSC	94402
w[*] TI{GFP[3xP3.cLa]=CRIMIC.TG4.1}CG43673[CR60378-TG4.1]	BDSC	94408
w[*]; TI{GFP[3xP3.cLa]=CRIMIC.TG4.1}Srg2[CR60387-TG4.1] dpr6[CR60387-TG4.1-X]/TM3, Sb[1] Ser[1]	BDSC	94413
w[*]; TI{GFP[3xP3.cLa]=CRIMIC.TG4.0}CG3104[CR60420-TG4.0]/CyO	BDSC	94438

TI{GFP[3xP3.cLa]=KozakGAL4}CG5254[CR70245-KO-kG4] w[*]/FM7h	BDSC	95006
y[1] w[*]; TI{GFP[3xP3.cLa]=CRIMIC.TG4.1}Dif[CR70340-TG4.1]/SM6a	BDSC	95043
y[1] w[*] TI{GFP[3xP3.cLa]=CRIMIC.TG4.1}ldd[CR70313-TG4.1]	BDSC	97159
y[1] w[*]; TI{GFP[3xP3.cLa]=CRIMIC.TG4.0}Cyt-b5[CR70361-TG4.0]/SM6a	BDSC	97162
y[1] w[*]; TI{GFP[3xP3.cLa]=CRIMIC.TG4.1}dve[CR70543-TG4.1]/SM6a	BDSC	97198
y[1] w[*]; TI{GFP[3xP3.cLa]=CRIMIC.TG4.0}Pka-R1[CR70548-KO-TG4.0]	BDSC	97356
y[1] w[*]; TI{GFP[3xP3.cLa]=SA-KozakGAL4}porin[CR70553-KO-kG4]/SM6a	BDSC	97360
yw; P{w[+mC]=pPGB}Df31[CB02104]	A. C. Spradling	651
yw; P{w[+mC]=pPGC}sm[CC00233]	A. C. Spradling	712
yw; P{w[+mC]=pPGC}pum[CC00479]	A. C. Spradling	722
yw; P{w[+mC]=pPGC}Zasp66[CC00858]	A. C. Spradling	730
yw; P{w[+mC]=pPGC}Vha26[CC01380]	A. C. Spradling	745
y[1] w[*] P{y[+t7.7]=nanos-phiC31\int.NLS}X; P{y[+t7.7]=CaryP}Msp300[attP40]	BDSC	79604
w[1118]; PBac{w[+mC]=WH}Tbce[f00024]/CyO	BDSC	18286
w[*]; sna[Sco]/CyO, S[*] bw[1]	BDSC	3198
P{y[+mDint2]=Crey}1b, y[1] M{vas-int.Dm}ZH-2A w[*]; sna[Sco]/CyO, P{w[+mC]=GAL4-Kr.C}DC3, P{w[+mC]=UAS-GFP.S65T}DC7	BDSC	80098
y[1] w[*]; Kr[If-1] wg[Sp-1]/CyO; P{w[+mC]=loxP(DH.1)}6	BDSC	80084

Recombinant DNA		
FlyFos024444(Muc68D[16941]::S000169_fly_pretag)::2XTY1-SGFP-V5-preTEV-BLRP-3XFLAGdFRT	Sarov *et al.* (2016)	ConstructID#35649249831002394_H07
Software and algorithms		
ImageJ (Fiji)	ImageJ	v1.54f
IMARIS	Oxford Instruments	v10.0.0
Premiere	Adobe	V22.64
VisiView	Visitron Systems	v6.0.0.18
Cell Ranger	10X Genomics	v6.0.1
R	The R Project	v4.1.2
R studio	PBC formerly RStudio	R studio
Seurat	Hao *et al.* (2021)	v4.3.0
Nextflow	Di Tommaso *et al.* (2017)	v22.10.1
DESeq2	Love, Huber, &Anders (2014)	v1.34.0
ggplot2	Wickham (2016)	v 2_3.4.3
Stellaris Probe Designer	Biosearch Technology Inc.	https://www.biosearchtech.com/support/tools/design-software/stellaris-probe-designer
Other		
µ-Dish 35 mm low	ibidi	Cat#80136
20 µm syringe	BD	Cat#340595

Supplemental figures

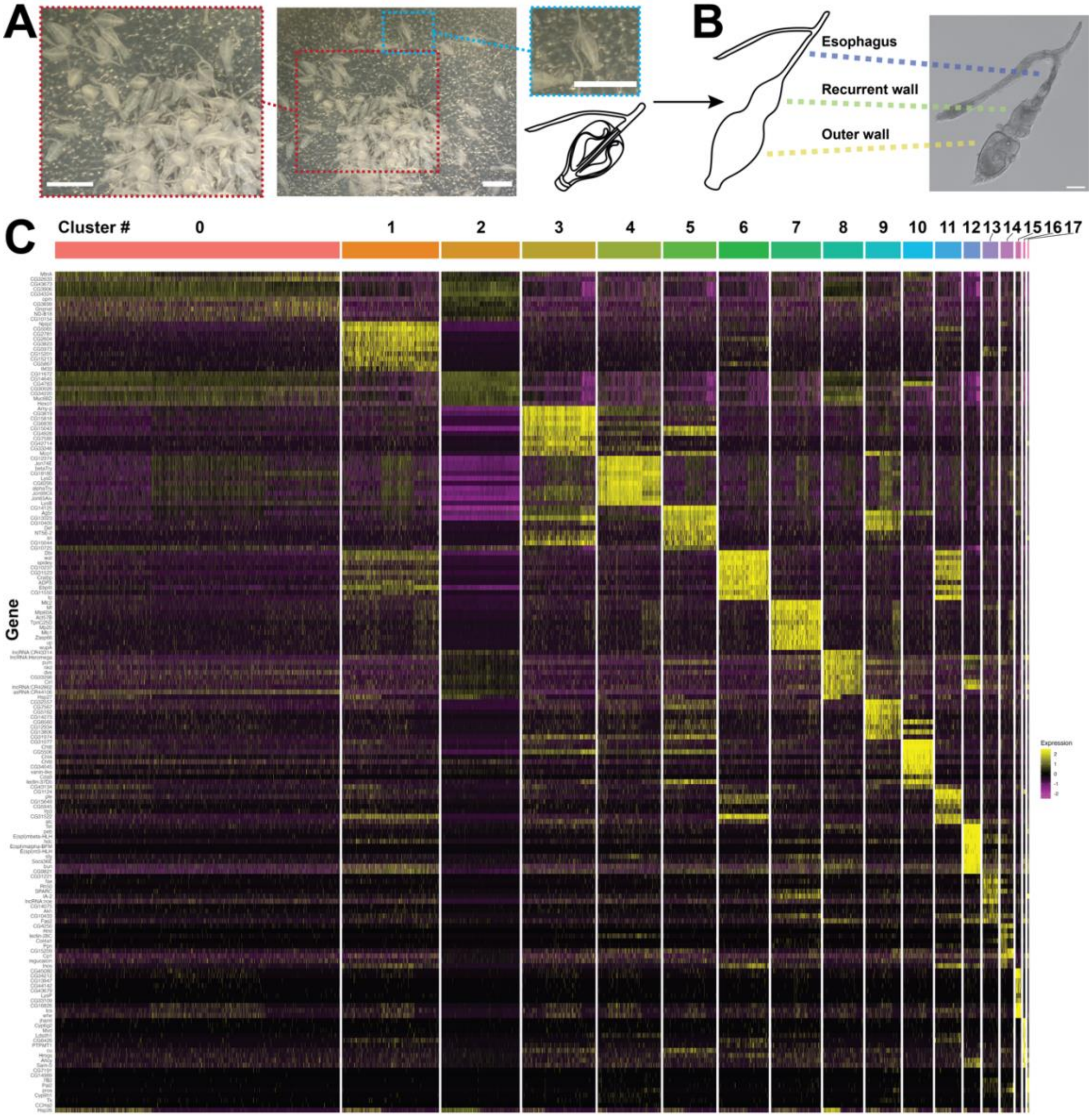

Figure S1. Experimental methods and initial analyses supporting scRNAseq of the Drosophila foregut.

A. Dissected PV tissue used for scRNAseq (center), magnified at left and right. B. Diagrams showing the unfolding of a triple-layer PV epithelium to facilitate uniform cell recovery for scRNAseq. The micrograph at right shows unfolded tissue with three PV

layers exposed. C. Preferential gene expression within 18 scRNAseq initial clusters (#0-17 at top) analyzed to display cluster-specific genes. Scale bars = 500 µm (A); and 100 µm (B).

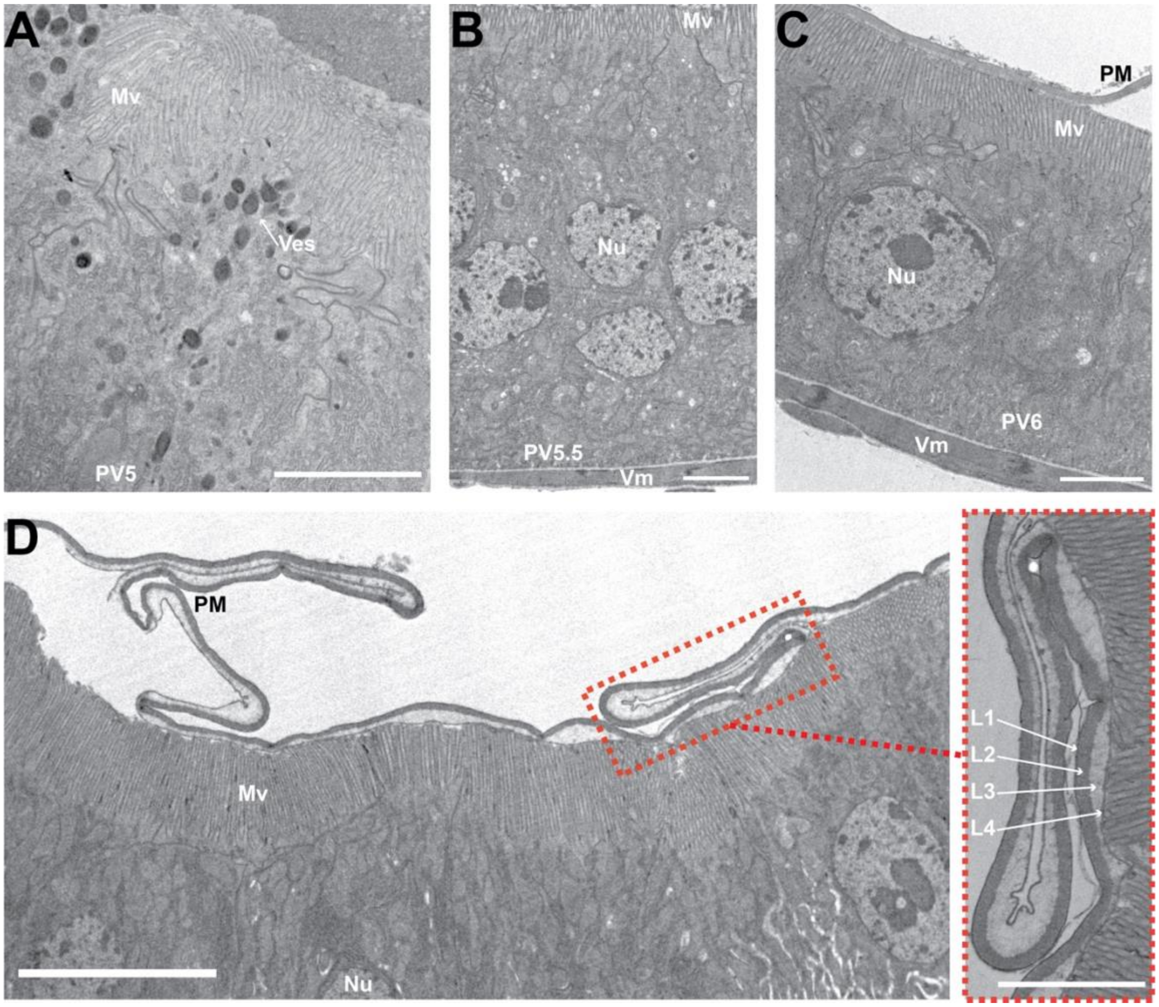

Figure S2. Electron microscopical analysis reveals PV outer wall cells and the PM. A-C. PV5 cells exhibit apical microvilli with secretory granules docking nearby for secretion (A). The microvilli morphology changes as the tissue moves posterior to PV5.5 (B) and PV6 (C) cells. D. The structurally completed PM formed at the posterior PV with four layers resolved by electron microscopy. The PM shows buckling as the intestinal lumen becomes narrower from anterior to posterior PV. Peritrophic matrix (PM), microvilli (Mv), secretary vesicle (Ves), and nucleus (Nu). Scale bars = 2 µm (A-C); 5 µm (L in D); and 2 µm (R in D).

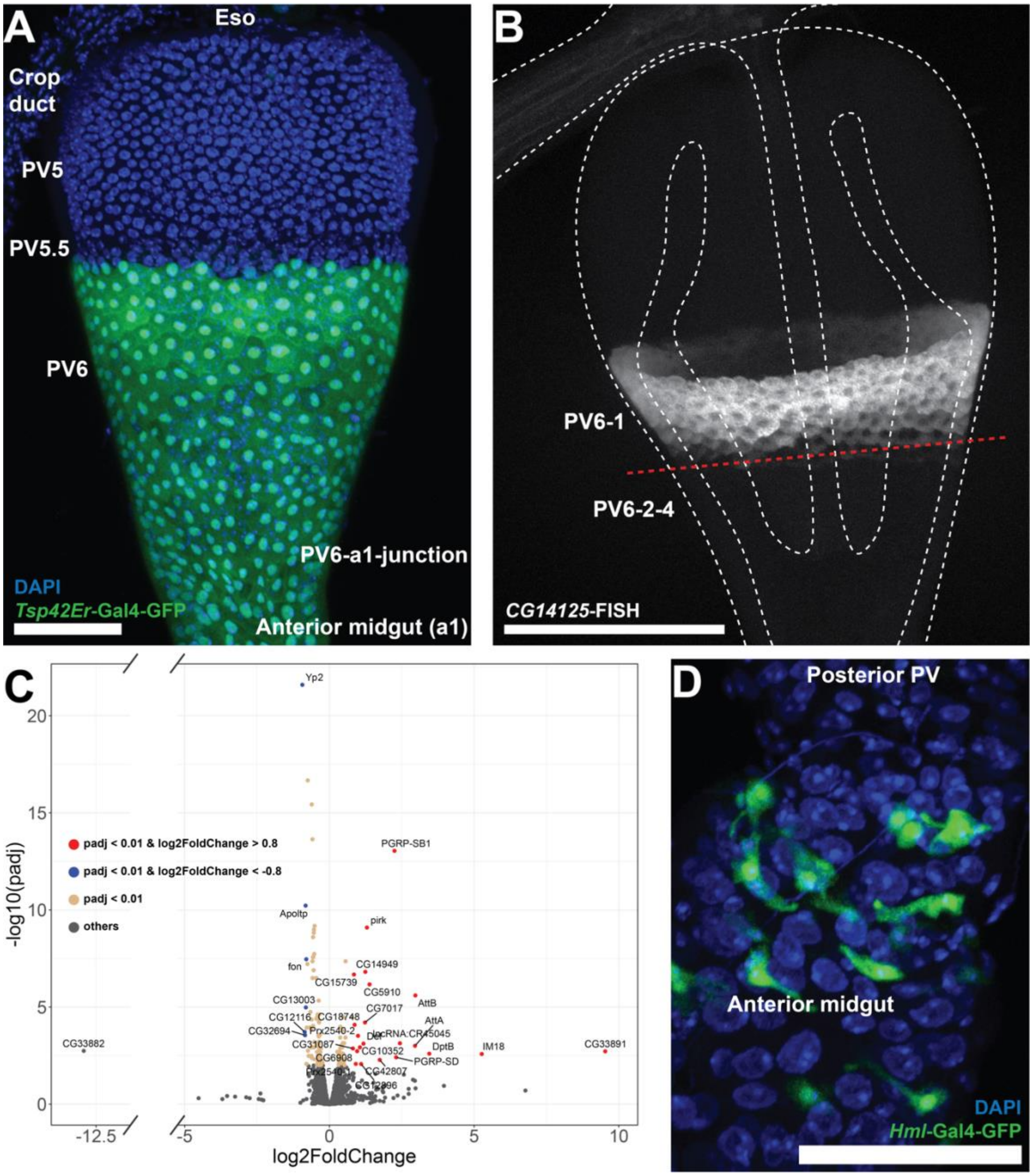

Figure S3. Foregut tissue exhibits various sources of immunity.

A. The posterior PV outer wall and anterior 1 (a1) midgut junctional region highlighted by *Tsp42Er*-Gal4-GFP expresses. B. PV6-1 cells highlighted by *CG14125* RNA-FISH. C. Volcano plot showing differentially expressed genes in PV tissues of adult flies reared in the presence of one normal gut microbiome species, *Lactiplantibacillus plantarum*

(*LpWF*) compared to adult flies reared axenically. D. *Hml*-Gal4-GFP reveals putative hemocytes in the posterior PV and anterior midgut junction. Scale bars = 50 µm (A); 100 µm (B); and 50 µm (D).

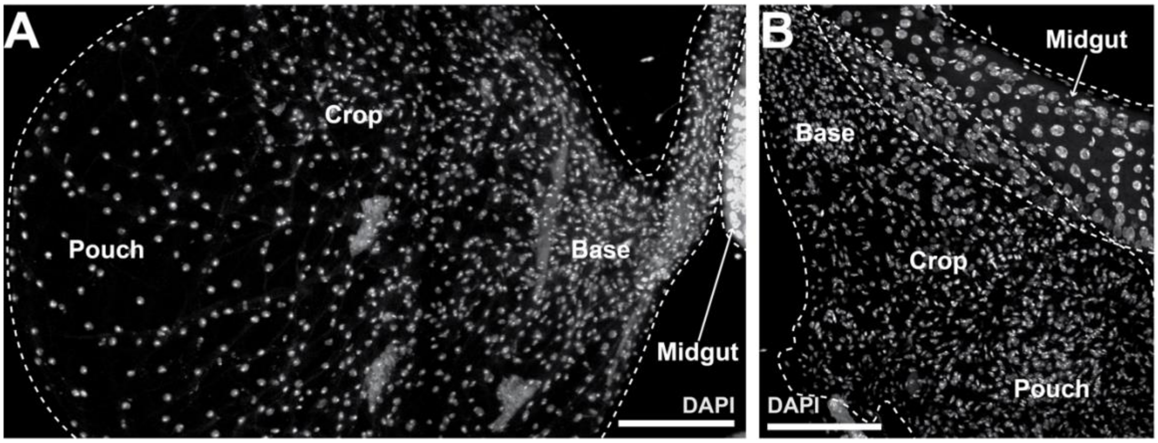

Figure S4. The crop expands supporting transient food storage.

Adult Drosophila develops crop structures, a muscular sack, to store food ingestion transiently by expanding the crop pouch significantly (A) compared to its shrunk state (B). Scale bars = 100 µm.

Supplemental legends

Dataset S1. PV cell types and numbers.

Data of PV cell quantification using the microscopical analyses and IMARIS spot function (IMARIS_rep#) as well as cell quantification using scRNAseq (SC_rep#) are given.

Dataset S2. Reporter lines used to refine foregut cell types.

Categories include Stock ID; Stock origin (BDSC, Bloomington Drosophila Stock Center; ACS, Spradling lab stock); Genotype; Targeted gene; and Type (Gal4 lines including CRIMIC_Gal4, RMCE_Gal4, GDP_Gal4, InSITE_enhancer_trap, Enhancer_trap, Gal4, and Janelia_Gal4; and GFP tag lines including RMCE_protein_trap, Carnegie_protein_trap, modERN_protein_trap, and Protein_trap).

Dataset S3. Gene expression of 19 foregut cell clusters by scRNAseq.

The Excel data file shows average normalized expressions of individual genes within individual resolved cell clusters. The 19 cell clusters include visceral muscle, esophagus, proventriculus 1 (PV1), proventriculus 2 (PV2), proventriculus 3 (PV3), proventriculus 4 (PV4), proventriculus 5 (PV5), proventriculus 5.5 (PV5.5), proventriculus 6-1 (PV6-1), proventriculus 6-2 (PV6-2), proventriculus 6-3 (PV6-3), proventriculus 6-4 (PV6-4), growing cell, *corpus allatum* (CA), *corpus cardiacum* (CC), neuronal-1, neuronal-2, garland cell nephrocyte (garland cell), and hemocyte-like immune cells (hemocyte-like).

Dataset S4. Preferential gene expression of 19 foregut cell clusters by scRNAseq.

Each tab of the Excel data file shows the genes preferentially expressed within individual cell clusters including visceral muscle, esophagus, proventriculus 1 (PV1), proventriculus

2 (PV2), proventriculus 3 (PV3), proventriculus 4 (PV4), proventriculus 5 (PV5), proventriculus 5.5 (PV5.5), proventriculus 6-1 (PV6-1), proventriculus 6-2 (PV6-2), proventriculus 6-3 (PV6-3), proventriculus 6-4 (PV6-4), growing cell, *corpus allatum* (CA), *corpus cardiacum* (CC), neuronal-1, neuronal-2, garland cell nephrocyte (garland cell), and hemocyte-like immune cells (hemocyte-like). Other table matrices include the average log2 fold difference of a gene expression in a particular cluster compared to the remaining cells (Avg_log2FC), its associated p-value (P_val), and p-adjusted value (P_val_adj).

Dataset S5. Cell type specific gene expression and associated transgenic lines identified.

Each tab of the Excel data file shows the genes specifically expressed within individual cell clusters and associated transgenic lines. The cell clusters include visceral muscle, esophagus, proventriculus 1 (PV1), proventriculus 2 (PV2), proventriculus 3 (PV3), proventriculus 4 (PV4), proventriculus 5 (PV5), proventriculus 5.5 (PV5.5), proventriculus 6-1 (PV6-1), proventriculus 6-2 (PV6-2), proventriculus 6-3 (PV6-3), proventriculus 6-4 (PV6-4), growing cell, *corpus allatum* (CA), *corpus cardiacum* (CC), neuronal-1, neuronal-2, garland cell nephrocyte (garland cell), and hemocyte-like immune cells (hemocyte-like). Other table matrices include average log2 fold difference of a gene expression in a particular cluster comparing to the remaining cells (Avg_log2FC), its associated p-adjusted value (P_val_adj), its expression level in the most abundantly expressed cluster to it in the second most abundantly expressed cluster (Cluster_specificity), notable expression in other cluster(s), as well as associated transgenic lines including CRIMIC and REMC lines.

Dataset S6. Gene expression in adult fly crop and salivary gland tissues by RNAseq.

The Excel data file shows normalized expressions (TPM) of all genes from RNAseq of the adult fly crop (Crop_rep#) and salivary gland (SG_rep#) tissues.

Dataset S7. Differential gene expression analysis on PV tissues of adult flies reared in the presence of *LpWF* compared to adult flies reared axenically.

The Excel data file shows differentially expressed genes (Log2FoldChange > 0.8, Padj < 0.01) of the PV tissues upon exposure to a normal commensal bacterial species, *Lactiplantibacillus plantarum* (*LpWF*) compared to the axenic controls. Other table matrices include mean expression level (BaseMean), log2 fold difference of a gene expression (Log2FoldChange), standard error of the log2FoldChange estimate (LfcSE), Wald statistic (Stat), p-value (Pvalue), and p-adjusted value (Padj).

Dataset S8. Table for sequence information.

The table contains sequence information of the RNA-FISH probes and the *GFP* CDS used for the scRNAseq analysis.

Movie S1. Movements of a freshly dissected proventriculus cultured *in vitro*.

SI References

P. Di Tommaso, M. Chatzou, E. W. Floden, P. P. Barja, E. Palumbo, & C. Notredame, Nextflow enables reproducible computational workflows. *Nature biotechnology*, **35(4)**, 316–319 (2017).

D. Li-Kroeger, O. Kanca, P. T. Lee, S. Cowan, M. T. Lee, M. Jaiswal, J. L. Salazar, Y. He, Z. Zuo, & H. J. Bellen, An expanded toolkit for gene tagging based on MiMIC and scarless CRISPR tagging in *Drosophila*. *eLife*, **7**, e38709 (2018).

M. Marchetti, C. Zhang, & B. A. Edgar, An improved organ explant culture method reveals stem cell lineage dynamics in the adult *Drosophila* intestine. *eLife*, **11**, e76010 (2022).

M. Sarov, C. Barz, H. Jambor, M. Y. Hein, C. Schmied, D. Suchold, B. Stender, S. Janosch, V. V. K J, R. T. Krishnan, A. Krishnamoorthy, I. R. Ferreira, R. K. Ejsmont, K. Finkl, S. Hasse, P. Kämpfer, N. Plewka, E. Vinis, S. Schloissnig, E. Knust, V. Hartenstein, M. Mann, M. Ramaswami, K. VijayRaghavan, P. Tomancak, F. Schnorrer, A genome-wide resource for the analysis of protein localization in Drosophila. *eLife*, **5**, e12068 (2016).
